# An Ideal PPAR Response Element Bound to and Activated by PPARα

**DOI:** 10.1371/journal.pone.0134996

**Published:** 2015-08-04

**Authors:** John Tzeng, Jaemin Byun, Ji Yeon Park, Takanobu Yamamoto, Kevin Schesing, Bin Tian, Junichi Sadoshima, Shin-ichi Oka

**Affiliations:** 1 Department of Cell Biology and Molecular Medicine, Rutgers Biomedical Health Sciences, Newark, NJ 07103, United States of America; 2 Department of Biochemistry and Molecular Biology, Rutgers Biomedical Health Sciences, Newark, NJ 07103, United States of America; Nihon University School of Medicine, JAPAN

## Abstract

Peroxisome proliferator-activated receptor-α (PPARα), a nuclear receptor, plays an important role in the transcription of genes involved in fatty acid metabolism through heterodimerization with the retinoid x receptor (RXR). The consensus sequence of the PPAR response element (PPRE) is composed of two AGGTCA-like sequences directionally aligned with a single nucleotide spacer. PPARα and RXR bind to the 5’ and 3’ hexad sequences, respectively. However, the precise sequence definition of the PPRE remains obscure, and thus, the consensus sequence currently available remains AGGTCANAGGTCA with unknown redundancy. The vague PPRE sequence definition poses an obstacle to understanding how PPARα regulates fatty acid metabolism. Here we show that, rather than the generally accepted 6-bp sequence, PPARα actually recognized a 12-bp DNA sequence, of which the preferred binding sequence was WAWVTRGGBBAH. Additionally, the optimized RXRα hexad binding sequence was RGKTYA. Thus, the optimal PPARα/RXRα heterodimer binding sequence was WAWVTRGGBBAHRGKTYA. The single nucleotide substitution, which reduces binding of RXRα to DNA, attenuated PPARα-induced transcriptional activation, but this is not always true for PPARα. Using the definition of the PPRE sequence, novel PPREs were successfully identified. Taken altogether, the provided PPRE sequence definition contributes to the understanding of PPARα signaling by identifying PPARα direct target genes with functional PPARα response elements.

## Introduction

Nuclear receptors are ligand-activated transcription factors that govern nutrient- and hormone-mediated responses[1]. The activating ligands include fatty acids, vitamins, bile acids, sterols, and hormones. Nuclear receptors sense the nutritional and hormonal status and promote transcription of the target genes necessary to produce a biological response. The functional entities of nuclear receptors include monomers, homodimers, and heterodimers. All nuclear receptors recognize and bind to a hexad AGGTCA-like sequence as a monomeric unit. The response element for dimer entities of nuclear receptors is composed of 2 hexad sequences, which can be configured into direct, invert, and evert repeats[2]. Although nuclear receptors mediate their functions primarily through DNA binding, for many nuclear receptors, the substantive DNA binding site sequence has not yet been fully determined.

Peroxisome proliferator-activated receptor-α (PPARα) is a member of the PPAR nuclear receptor subfamily, which also includes PPARβ/δ and PPARγ [3]. All PPARs play important roles in fatty acid metabolism. In particular, PPARα is activated by a fatty acid ligand and transcribes genes involved in fatty acid catabolism, thereby promoting fatty acid clearance. Fibrates are pharmaceutical ligands for PPARα and are used clinically for lowering plasma lipids in patients with hyperlipidemia. Loss of PPARα in mice results in obesity and increased serum lipid derivatives such as triglyceride, phospholipids, and cholesterol[4]. These abnormalities are most likely caused by impaired fatty acid catabolism/clearance.

PPARα forms a heterodimer with Retinoid X receptor (RXR) and together they bind to their DNA binding element, called PPAR response element (PPRE)/Direct Repeat 1 (DR1)[5]. The consensus sequence of PPRE/DR1 is composed of 2 core hexad sequences directionally aligned and separated by a single nucleotide spacer (AGGTCANAGGTCA, where N is any nucleotide)[6], and PPARα and RXR bind to the 5’ and 3’ AGGTCA sequences, respectively. In general, the consensus sequence of a transcription factor binding element is defined as comprising the most frequently observed nucleotide at each position in a sequence alignment. Interestingly, none of the endogenous PPREs thus far identified possesses the consensus sequence. Rather, the majority of actual PPREs represent degenerate sequences rather than the consensus AGGTCA-N-AGGTCA, with examples including *CD36* (AAGTCA-G-AGGTCA), *Cpt1b* (AGGGAA-A-AGGTCA), *Fatp1* (AGGGCA-C-AGGAGA), *Txnip* (AGGACA-G-AGGGGG), and *Cidea* (GGGGGA-A-AGGTTA). Thus, the PPARα/RXR heterodimer is capable of binding to and activating transcription through redundant sequences, but the degree of acceptable sequence variability in the PPRE is unknown. Compared to the large number of genes known to be controlled by PPARα, functional PPREs have been identified in only a limited number of target genes[7]. The vague definition of the PPRE sequence is an obstacle to identifying PPARα direct target genes harboring functional PPREs.

In this paper, we performed a comprehensive characterization of the DNA sequence of the PPRE for PPARα. The biotin-labeled double-stranded DNA pull-down assay was applied to determine the optimized DNA sequence for PPARα and RXRα binding. The effect of each position of the PPRE sequence in the PPARα-induced transcription was examined by reporter gene assays in primary cultured cardiac myocytes and H9c2 cells derived from rat cardiac myoblasts, since the pathophysiological significance of PPARα has been well-demonstrated in the heart and we have a long-standing interest in the role of PPARα in cardiac pathophysiology. Our data indicated that, rather than the generally accepted 6 bp of the AGGTCA sequence, PPARα was able to recognize a 12-bp sequence, composed of the core hexad sequence, a spacer, and 5 bp of the 5’ extended sequence. The optimized PPARα binding sequence was WAWVT-RGGBBA-H (W: A and T; V: A, G, and C; R: A and G; B: G, C, and T; H: A, C, and T), while RGKTYA was the optimized RXRα core hexad binding sequence (K: G and T; Y: T and C). Unexpectedly, nucleotide substitution at several positions, which reduces PPARα DNA binding, enhanced PPARα-induced transcriptional activation. Using the definition of the PPRE sequence, novel PPREs were successfully identified. Thus, the PPRE sequence provided by this study can contribute to identifying PPARα direct target genes with the functional PPREs, which will accelerate our understanding of how PPARα governs fatty acid metabolism.

## Materials and Methods

### Biotin-labeled DNA pull-down

A master mixture of the recombinant proteins (0.1 to 0.5 μg/150 μl) and biotin-labeled DNAs (1 pMol/150 μl) were prepared with binding buffer [10 mM Hepes pH 7.9, 2.5 mM MgCl_2_, 50 mM KCl, 150 mM NaCl, 5% glycerol, 1 mM DTT, 0.1% IGEPAL CA-630]. The master mixture was dived into 1.5 ml tubes (150 μl/tube). Then streptavidin-beads (15 μl (50% slurry)/150 μl) and 10- and 30-fold excessive unlabeled competitor DNAs (10 to 30 pMol) were added. After 2 hours of rotation, the recovered proteins were washed 3 times with 1 ml of binding buffer and subjected to Western blot analyses with anti-PPARα (Cayman, #101710) and anti-RXRα (Santa Cruz, ΔN197, sc-774) antibodies. For incubation with WY14,643, 1 μM WY14,643 was added to the binding buffer used for the initial incubation and washing. The signal density of Western blot analyses was quantified with the ImageJ program. The signal density without a competitor was defined as 100.

### Cell Culture

The primary cultured myocytes were cultured with complete medium containing Dulbecco’s modified Eagle medium/F12 supplemented with 5% horse serum, 4 μg/ml transferrin, 0.7 ng/ml sodium selenite, 2 g/l bovine serum albumin (fraction V), 3 mM pyruvate, 15 mM Hepes pH 7.1, 100 μM ascorbate, 100 mg/l ampicillin, 5 mg/l linoleic acid, and 100 μM 5-bromo-2’-deoxyuridine (Sigma). The H9c2 and Cos7 cells were cultured with Dulbecco’s modified Eagle medium (DMEM) containing 10% Fetal Bovine Serum.

### Luciferase assay

Luciferase assays were performed in primary cultured rat cardiac myocytes and H9c2 cells, a rat cardiac myoblast cell line, and Cos7, fibroblast-like cells. For cultured myocytes, the medium was exchanged to serum-free DMEM/F12 medium after 24 hours of isolation. Reporter plasmids (0.3 μg per well) were transfected into the cells plated on a 12-well plate using LipofectAmine 2000 (Invitrogen). After 16 hours of transfection, the cells were incubated with 0.01, 0.1, and 1 μM WY14,643 for 6 hours, and then the luciferase assays were performed with a luciferase assay system (Promega). Ethanol was used as a vehicle control, because the WY14,643 was solved with ethanol. For overexpression of PPARα-induced reporter gene activation, 0.3 μg of reporter and 0.1, 0.3 and 0.7 μg of PPARα expression vector (pDC316-PPARα) were transfected into the H9c2, Cos7 cells or myocytes (12 well plate) using LipofectAmine 2000 (Invitrogen). Total plasmids were kept at 1 μg-per-well with pDC316 control vector. Luciferase assays were performed 2 to 3 days after transfection. For WY14,643 treatment in Cos7 cells, 0.3 μg of reporter and 0.1 μg of pDC316-PPARα was transfected to the cells. After 2 to 3 days of transfection, the cells were incubated with 0.01, 0.1, and 1 μM WY14,643 for 6 hours, and then the luciferase assays were performed.

### Plasmids

The minimal promoter luciferase reporter was generated by insertion of the minimal promoter sequence (CTCGAGTAGAGGGTATATAATGGAAGCTCGACTTCCAG) between the XhoI and HindIII sites of pGL3Basic (Promega). The artificial PPRE sequences were inserted between NheI and XhoI to generate a series of reporter constructs. The reporter gene constructs for intrinsic promoters were generated by insertion of approximately 500 bp of the promoter region into pGL3Basic. The G to A mutation in the reporter gene constructs of the *Acot2* and *Fbp2* promoters was performed by standard site-directed mutagenesis. The mammalian expression vector for PPARα (pDC316-PPARα) and bacterial expression vector for GST-fused PPARα (pCold-GST-PPARα) were described previously[8]. The bacterial expression vector for GST-fused RXRα (pCold-GST-RXRα) was generated by insertion of the RXRα cDNA into pCold-GST. The GST was fused to the N-terminal of PPARα and RXRα.

### Recombinant proteins

The BL21 *E*.*coli* strain was transformed with pCold-GST-PPARα and pCold-GST-RXRα. The *E*.*coli* were grown in 3 ml LB medium overnight at 37°C and then transferred to 250 ml LB medium. When the optical density at 600 nm reached 0.5 to 1, the temperature was reduced to 15°C, and Isopropyl β-d-1 thiogalactopyranoside was added (100 μM final concentration) 1 hour later. After overnight culture at 15°C, the *E*.*coli* were lysed in lysis buffer (1% Triton X-100, 1 mM DTT, PBS) with sonication. The lysate was incubated with 0.5 ml Glutathione-sepharose 4B (GE Healthcare) for 1 hour at 4°C. The sepharose was washed 3 times with 5 ml lysis buffer, and then suspended with 1 ml cleavage buffer (20 mM Tris pH 7, 150 mM NaCl, 1 mM DTT) containing 200 units/ml of PreScission protease (GE Healthcare). After overnight incubation at 4°C, the supernatant containing the recombinant proteins was collected. The recombinant proteins were stored at -80°C.

### Bioinformatics screening for PPRE in mouse genome

The mouse genome (mm9) was screened for the provided PPRE sequence within +/- 2 kb of transcription starting sites. The PPRE sequence that we used for the search was DAWVT-RGGBBA-N-RGKTBA, while allowing 1 non-preferred nucleotide in the last 3 bp of the 5’ hexad element and spacer positions.

### Animals

C57BL/6 and 129Sv mixed background PPARα knockout mice were obtained from the Jackson Laboratory [9,10]. PPARα knockout mice were backcrossed to FVB. F2 (75% FVB) and F3 (82.5% FVB) mice were used for this study. Cardiac-specific PPARα overexpression (Tg-PPARα) mice, line 404–3, on an FVB background were kindly provided by Drs. Daniel P. Kelly and Teresa Leone at Sanford-Burnham Medical Research Institute[11]. The breeding cages for these mice were maintained in a specific pathogen-free animal facility. The obtained mice were transferred to and housed in a conventional animal facility after weaning. In either facility, animals were housed under a 12 hours light/12 hours dark cycle with free access to a normal rodent diet and water. The room temperature was kept at 18–23°C. Both genders of 2-8-month-old mice were used. The body weights (Mean±SD (g)) of PPARα knockout and the control littermates were 27.8±5.1, and Tg-PPARα and the control littermates were 25.8±3.6. The number of mice used for each study was calculated with Power and Sample Size Calculation (http://biostat.mc.vanderbilt.edu/wiki/Main/PowerSampleSize) with the following inputs; design: independent, α: 0.05, Power: 0.8, and m: 1 or 2. The input values of δ and σ were varied based upon the expected difference and standard deviations. All procedures involving animals were performed in accordance with protocols approved by the Institutional Animal Care and Use Committee (IACUC) of Rutgers Biomedical and Health Sciences (Protocol Number: 11134A3D061).

### Echocardiography

Mice were anesthetized using 12 μl/g of body weight of 2.5% Avertin (Sigma), and echocardiography was performed using ultrasonography (Acuson Sequoia C256, Siemens Medical Solutions USA Inc., Malvern, PA). A 13-MHz linear ultrasound transducer was used. Two-dimension guided M-mode measurements of left ventricular (LV) internal diameter were taken from at least three beats and averaged. LV end-diastolic diameter (LVEDD) was measured at the time of the apparent maximal LV diastolic dimension, while LV end-systolic diameter (LVESD) was measured at the time of the most anterior systolic excursion of the posterior wall. LV ejection fraction and fractional shortening were calculated as follows: Ejection fraction = [(LVEDD)^3^-(LVESD) ^3^]/(LVEDD)^3^ x 100; Fractional shortening = (LVEDD-LVESD)/LVEDD x 100.

### Fatty acid oxidation

Animals were euthanized by pentobarbital, 120mg/kg of body weight. Dissected ventricular tissue was washed with PBS and weighed. The tissue was homogenized in 400 μl [0.25 M Sucrose, 1 mM EDTA]/100 mg tissue weight. The heart homogenate (40 μl) was dispensed per tube and centrifuged at 800g for 1.5 minutes. The precipitates were incubated with 200 μl reaction buffer [10 mM Hepes pH 7.4, 150 mM KCl, 0.1 mM EDTA, 1 mM K_2_HPO_4_, 10 mM MgCl_2_, 1 mM Malate, 1 mCi/20 pmol ^3^H-Oleic acid] at 37°C for 10 minutes. The reactions were terminated by the addition of 1 ml chloroform/methanol (2:1). The samples were kept at -20°C for 2 to 24 hours. After centrifugation at 12,000g at room temperature for 10 minutes, the supernatants (approximately 400 μl) containing ^3^H_2_O were transferred to scintillation vials and mixed with 4 ml scintillation fluid. The radioactivity was measured with a scintillation counter.

### Microarray analyses

The accession number of the microarray data used for this study is GSE33101 at the Gene Expression Omnibus.

### Quantitative RT-PCR

Total RNA was prepared from left ventricles using the RNeasy Fibrous Tissue Mini Kit (Qiagen), and then cDNA was generated using M-MLV Reverse transcriptase (Promega). Real-time RT-PCR was performed using the Maxima SYBR Green qPCR master mix (Fermentas). β-actin was used as an internal control. The mean value from wild-type mice was expressed as 1. PCRs were carried out using the following oligonucleotide primers (5’ to 3’): *Abcd2*: ATATTTCAGGCTGCTATTGGGGCTG and AGTGTCCAACTGTTCAAAGCGCCA; *Acox1*: ATGAATCCCGATCTGCGCAA and TTCTCGATTTCTCGACGGCG; *Cd36*: TTTGGGAACCACATCCGCCAA and TTATGCCTGTGAGCTGGCCAC; *Cpt2*: GGCCAGCTGACCAAAGAAGCAG and GGTGGACAGGATGTTGTGGTTTATC; *Ech1*: CTGACGAGGCCCTGGACAGT and TGATTTTTGACCCCTGCACAGCCA; *Fabp3*:AAGGAGGCGTGACCTGGCTG and ACCTTGGAGCACCCTTTGGATACA; *Fabp4*: AGACGACAGGAAGGTGAAGAGC and CTCTTGTGGAAGTCACGCCTTTC; *Fatp1*: ATCTTCCTGCGTCTTCTGCCCC and TGCGGGCATGGACTCTCTCATC; *Lpl*:AGTGAAAGCCGGAGAGACTCAGA and GACTTCTTCAGAGACTTGTCATGGCATT; *Mcad*:GAAGCTGATGAGGGACGCCA and GCTTGGAGCTTAGTTACACGAGG; *PPARα*: CTGGTCACGGAGCATGCGCA and TCTGTGCAAATCCCTGCTCTCCT; *PPARβ/δ*: TTGCTGCACCCCCTGCTCCA and GGTCCTCTGAACAGTCCGTGG; *PPARγ*: AGCTACTGCATGTGATCAAGAAGAC and CCCTCAAAATAATAGTGCAATCAATAGAAGG; *Vldlr*: GCCTGGGCCATCCTTCCTCTC and GTATGTGTGTCCTACAGAAGCGC; and β-actin: AAGACCTCTATGCCAACACAGTGC and CACTTGCGGTGCACGATGGAG. To determine the relative copy numbers of PPAR isoforms in the heart, each isoform-specific standard (amplicons) was used.

### Statistical methods and error bars

Statistical comparisons were made using the Student’s *t* test. *P*<0.05 was defined as statistically significant and indicated by a filled asterisk. *P*>0.05 was indicated by NS. All error bars represent S.E.M.

## Results

### A putative consensus sequence of the PPRE for PPARα

**Fig 1:**
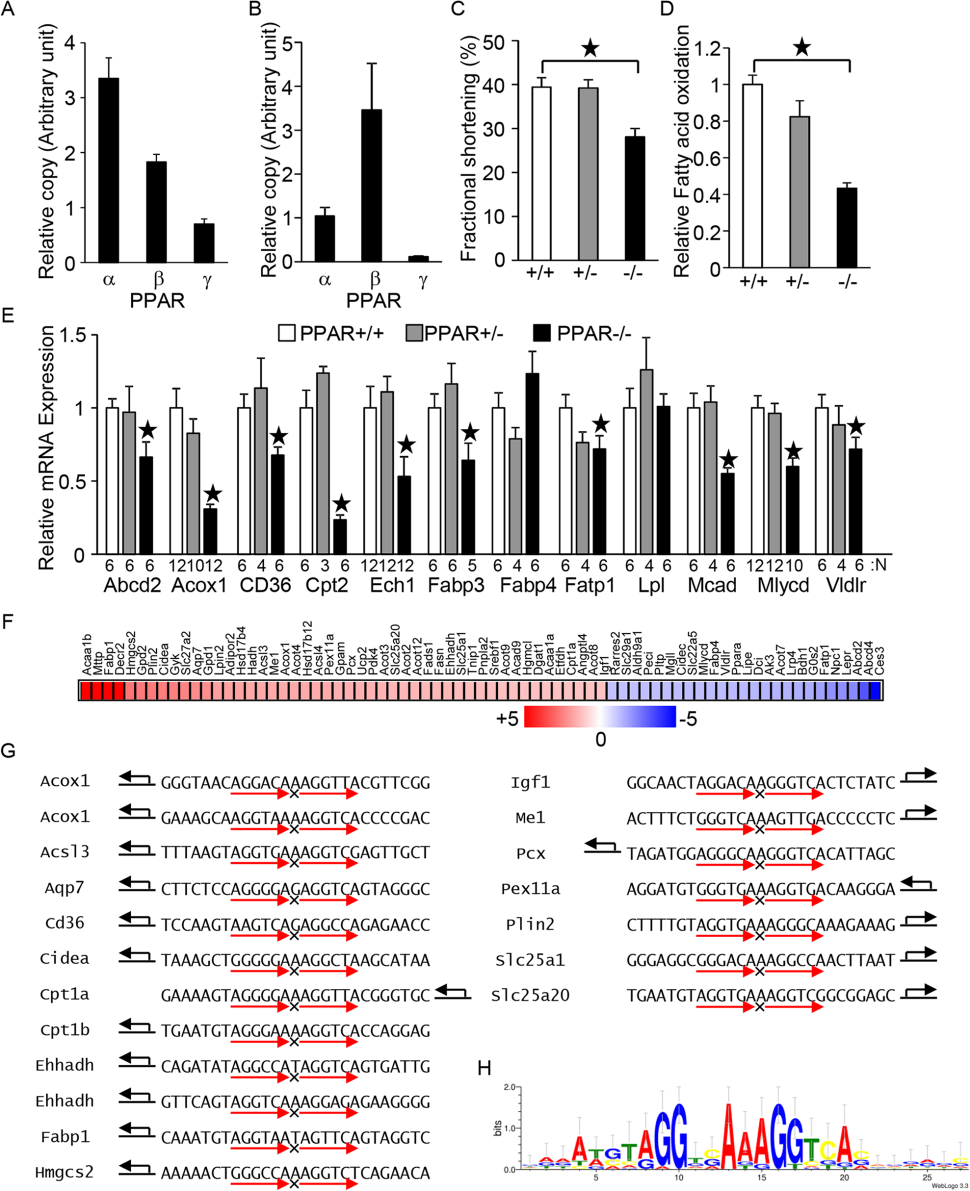
The putative PPARα response element in the heart. (A-B) PPARα is a major PPAR isoform in the heart. Relative copy numbers of PPAR isoforms at the mRNA level in FVB background mice (A) and in primary cultured neonatal cardiac myocytes (B) were examined by qPCR with isoform-specific PCR primers (n = 12 (A) and 5 to 6 (B)). (C) PPARα is required for cardiac systolic function at baseline. Echocardiographic measurements were performed on the indicated mouse genotypes. The cardiac systolic function was evaluated by determination of fractional shortening (n = 16–19). (D) PPARα is required for cardiac fatty acid oxidation activity. Fatty acid oxidation activity was measured in PPARα knockout mice (n = 5–9). (E) PPAR target gene expression in PPARα knockout mice. The relative mRNA levels were examined by qPCR. Both sexes of 2- to 6-month-old mice were used. The numbers of mice examined in each experimental group were indicated. (F) Relative PPAR target gene expression in Tg-PPARα mice. The heat map of PPAR target genes was generated according to the microarray results for NTg and Tg-PPARα. (G) Intrinsic PPRE sequences harbored by PPAR target genes upregulated in Tg-PPARα. The arrow indicates the transcription start site and direction of the gene body. The PPRE sequences were taken from published papers. (H) Putative PPRE consensus sequence in the heart. The PPRE sequence was generated using the PPREs shown in (G) with WebLogo [26].

The heart has been suggested to be an important target of the physiological functions of PPARs because the heart primarily utilizes fatty acids as an energy source[12]. To identify the major PPAR isoform in the adult heart and primary cultured neonatal cardiac myocytes, the relative copy numbers of PPAR mRNAs were examined by quantitative PCR. By this measure, of the 3 PPAR isoforms, PPARα appeared to be the most abundant in the adult heart (Fig 1A), whereas PPARβ/δ appeared to be the most abundant in the primary cultured myocytes (Fig 1B). To investigate the physiological significance of PPARα in the heart, cardiac contractility and PPAR target gene expression were examined in PPARα knockout (PPARα-/-) mice. PPARα-/- mice displayed impaired contractility, indicated by reduced fractional shortening (Fig 1C). The reduced fractional shortening is reported in 129/C57BL/6 mixed background PPARα-/- mice [13] but not in C57BL/6 background mice [14]. As expected, cardiac fatty acid oxidation declined in PPARα-/- mice (Fig 1D). In addition, many, if not all, PPAR target genes were downregulated in the PPARα-/- mice (Fig 1E). Thus, PPARα mediates physiological functions including fatty acid metabolism and cardiac myocyte contraction.

To generate the putative consensus sequence of PPRE, the expression levels of known PPAR target genes were examined in cardiac-specific PPARα overexpression (Tg-PPARα) mice. Microarray analyses revealed that many, if not all, PPAR target genes were upregulated in Tg-PPARα mice (Fig 1F). However, several PPARα target genes were downregulated, such as *Mlycd*, *Vldlr*, *G0s2*, and *Fatp1*. Known PPRE sequences found in the upregulated genes were aligned to generate the putative PPRE consensus sequence in the mouse heart (Fig 1G and 1H). The generated consensus sequence represents a perfect DR1 sequence (AGGTCANAGGTCA), although there is no individual PPRE that possesses this perfect DR1 sequence. In addition to the core hexad element, a sequence trend in approximately 6 bp of the 5’ extended region of the PPARα binding hexad element was observed, of which the most frequent nucleotides were represented by AAATGT. Furthermore, enrichment of the A spacer was observed in the putative PPRE/DR1 sequence, and the direction of the gene body frequently matched with the direction of the RXR binding site relative to PPARα.

### Effect of PPRE orientation

**Fig 2:**
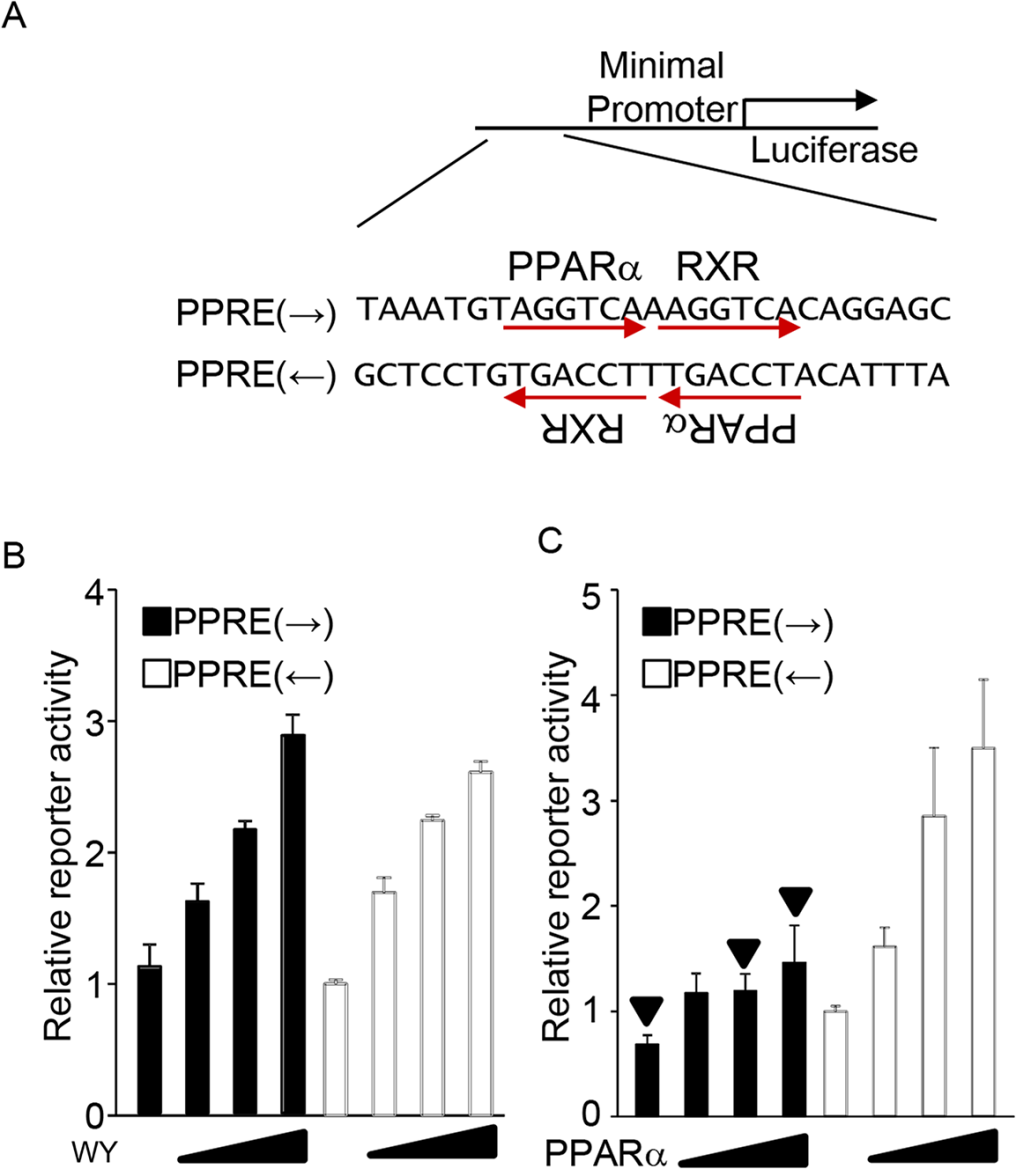
The effect of PPRE orientation. (A) Schematic representation of the reporter gene constructs. Both orientations of the PPRE consensus sequences indicated were connected to the luciferase gene with minimal promoter. PPARα and RXR binding hexad sequences are indicated. (B) Both orientations of PPRE relative to the luciferase gene were equally activated in primary cultured myocytes stimulated with WY14643 (n = 5–6). (C) Forced expression of PPARα preferentially activates the major direction of PPRE in H9c2 cells (n = 7–9). A solid triangle indicates statistical significance compared with PPRE(←) transfected with the same dose of PPARα expression vector.

To investigate the effect of the orientation of PPRE, a reporter assay was performed with a luciferase reporter construct driven by a 32-bp minimum promoter including the PPRE sequence with 5’ and 3’ 7-bp extended sequences, as shown in Fig 2A. Since PPARα is not significantly expressed in an immortalized cell line, we used primary cultured rat neonatal cardiac myocytes to examine the effect of the PPRE sequence on transcriptional activation induced by ligand-stimulated endogenous PPARα. At the same time, we used the H9c2 rat cardiac myocyte cell line to examine the effect of the PPRE sequence on transcriptional activation induced by exogenous PPARα overexpression to reduce animal usage. As shown in Fig 2B, both orientations of the PPRE were equally activated by WY14,647, an artificial ligand for PPARα, in primary culture cardiac myocytes. In contrast, overexpression of PPARα more strongly activated transcription when the orientation of the sequential PPARα and RXR binding site were opposite that of the luciferase gene body (Fig 2C). Thus, ligand-stimulated PPARα equally activates transcription in both directions, whereas PPARα-overexpression-induced transcriptional activation is sensitive to the orientation of the PPRE.

### Spacer nucleotide of PPRE

**Fig 3:**
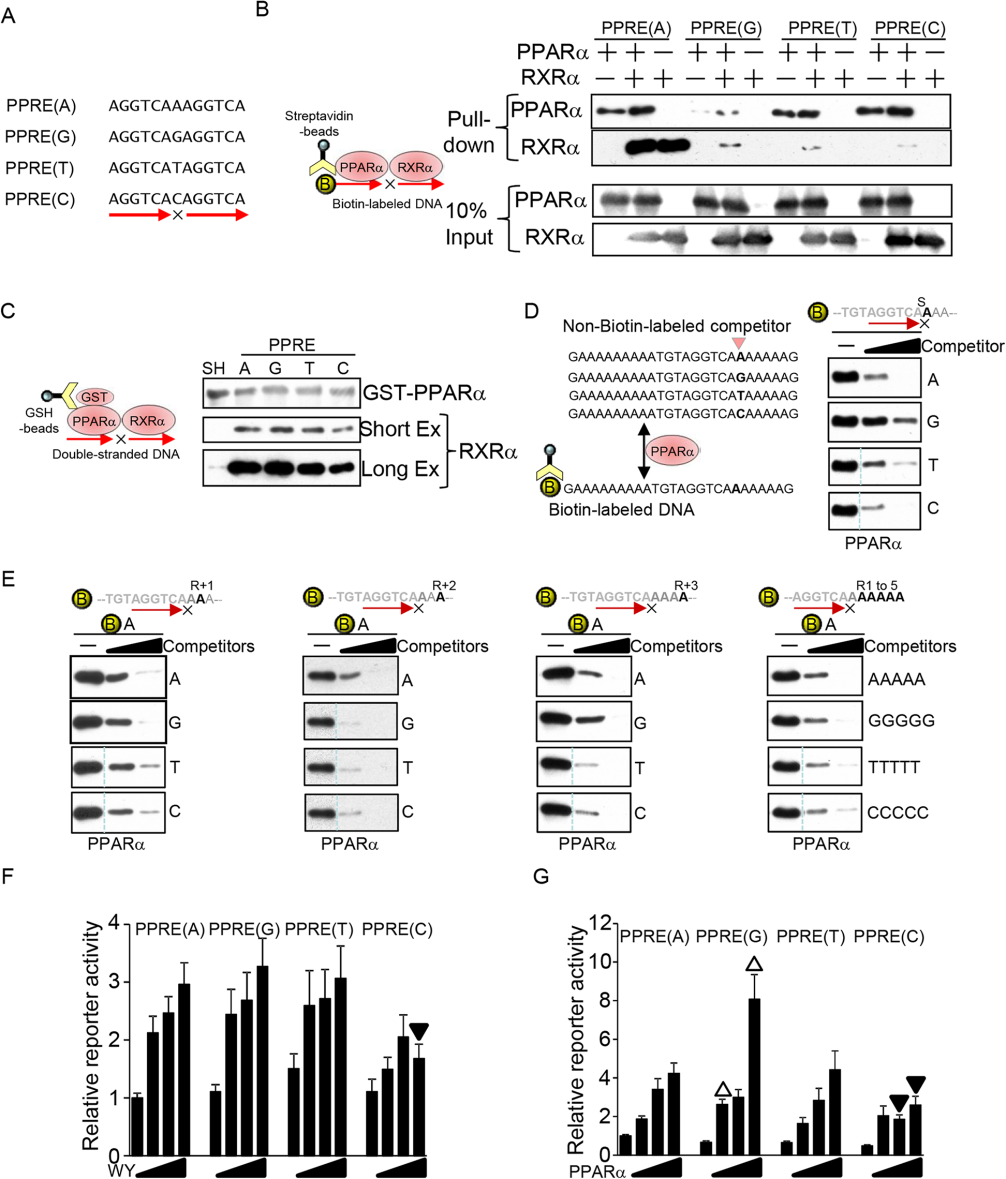
The effect of the spacer nucleotide in the PPRE. (A) Schematic representation of the double-stranded DNA used for *in vitro* binding and reporter gene assays. (B) The effect of spacer nucleotides in PPARα and RXRα binding to PPRE DNA. Biotin-labeled PPRE was incubated with recombinant PPARα and RXRα. (C) The effect of the spacer nucleotide within the PPRE on PPARα/RXRα heterodimer formation. GST-fused PPARα was incubated with RXRα and double-stranded DNA comprising PPREs possessing different spacer nucleotides. DNA comprising a single hexad element (AGGTCA-A-AAATCA) was used as a control (SH). Pull-down assays were performed with GSH beads. The Western blot analyses results with short- and long-time exposure are presented to show the reduced PPARα/RXRα heterodimerization in C spacer (Short Ex) and to show PPRE oligonucleotide-independent PPARα/RXRα heterodimerization (Long Ex), respectively. (D) The effect of spacer nucleotides on PPARα binding. The indicated biotin-labeled double-stranded DNA with A as a spacer was incubated with recombinant PPARα and unlabeled competitors. (E) The effect of the 3’ extended region of the PPARα binding hexad element in PPARα DNA binding. The indicated biotin-labeled double-stranded DNA was incubated with recombinant PPARα and unlabeled competitors (10- and 30-fold excess of biotin-labeled DNA). (D-E) All signals without competitors are identical among the 4 panels at each position. A dotted line indicates a discontinued signal but they are derived from identical blot/membrane. (F-G) The effect of a spacer nucleotide in PPARα ligand- (F) and PPARα-overexpression- (G) induced transcriptional activation. Minimal promoters with PPRE harboring different spacers were stimulated by the ligand and PPARα (n = 10–12). The triangle indicates statistical significance compared to PPRE(A) stimulated with the same dosage of WY14643 or transfected with the same amount of PPARα expression vector. Solid triangle: significantly reduced; open triangle: significantly enhanced. PPRE(A) is identical to PPRE(←) in Fig 2.

An enrichment of the A spacer was observed in the putative PPRE consensus sequence in the heart (Fig 1H). To investigate how the spacer nucleotide affects the DNA binding of PPARα and RXR, *in vitro* DNA binding assays using biotin-labeled double-stranded DNA were performed. The purity of the recombinant PPARα and RXRα is shown in S1 Fig. Single nucleotide substitution at the spacer position significantly affected the DNA binding of both PPARα and RXRα (Fig 3A and 3B). PPARα bound less effectively to the PPRE with a G spacer than to the others, and RXRα strongly bound to the PPRE with the A spacer even in the absence of PPARα, whereas RXRα DNA binding was PPARα-dependent when the spacer was not A. To examine the effect of the spacer nucleotide on PPARα/RXRα heterodimerization, recombinant RXRα was pulled down with GST-PPARα in the presence of double-stranded DNA comprising the PPRE with different spacer sequences. Although a PPRE with any spacer nucleotide enhanced heterodimer formation compared to the single hexad sequence (AGGTCAAAAATCA), the PPRE with C as the spacer displayed a slightly decreased ability to promote heterodimerization (Fig 3C). To investigate whether monomeric PPARα recognizes the spacer nucleotide, a biotin-labeled DNA pull-down assay was performed with DNA comprising the sequence of the putative PPRE in the heart (Fig 1H) but without the RXR binding half-site, because PPARα may bind to the RXR binding half-site AGGTCA element (Fig 3D Left). Unlabeled DNA with any of the four nucleotides at the spacer position competitively inhibited PPARα binding to biotin-labeled PPRE with an A spacer. Monomeric PPARα bound almost equally to the oligonucleotides with A, C, or T at the spacer position, whereas the oligonucleotide with G had a diminished ability to bind PPARα (Fig 3D, right). To test whether PPARα recognizes nucleotide positions other than the spacer, competition assays were performed using unlabeled DNA with each of the four nucleotides at the +1 to +3 positions in the RXR binding hexad element (R+1 to R+3) as competitors. As shown in Fig 3E, all nucleotides at positions R+1 to R+3 equally inhibited PPARα DNA binding. In addition, PPARα bound equally to all DNA sequences with repeated single nucleotides from R+1 to R+5. These results suggest that PPARα recognizes both the spacer position and its neighboring positions (Fig 3E). To investigate the effect of the spacer nucleotide upon transcriptional activation, a reporter gene assay was performed. As shown in Fig 3F and 3G, the PPRE with C as the spacer is less able to be activated by either PPARα ligand or PPARα overexpression. Although monomeric PPARα has less affinity for the PPRE with G as the spacer, transcriptional activation induced by the PPARα ligand was not attenuated compared to with other nucleotide spacers (Fig 3F). In addition, PPARα-overexpression-induced transcriptional activation was enhanced with the PPRE containing a G nucleotide spacer. On the other hand, the PPRE with a C nucleotide spacer had a relatively decreased ability to be activated by PPARα, which correlates well with its decreased ability to promote PPARα/RXR heterodimerization. Taken altogether, these results show that the spacer nucleotide affects PPARα and RXRα DNA binding and subsequent transcriptional activation.

### The optimized sequence of the 5’ extended region of the PPRE

**Fig 4:**
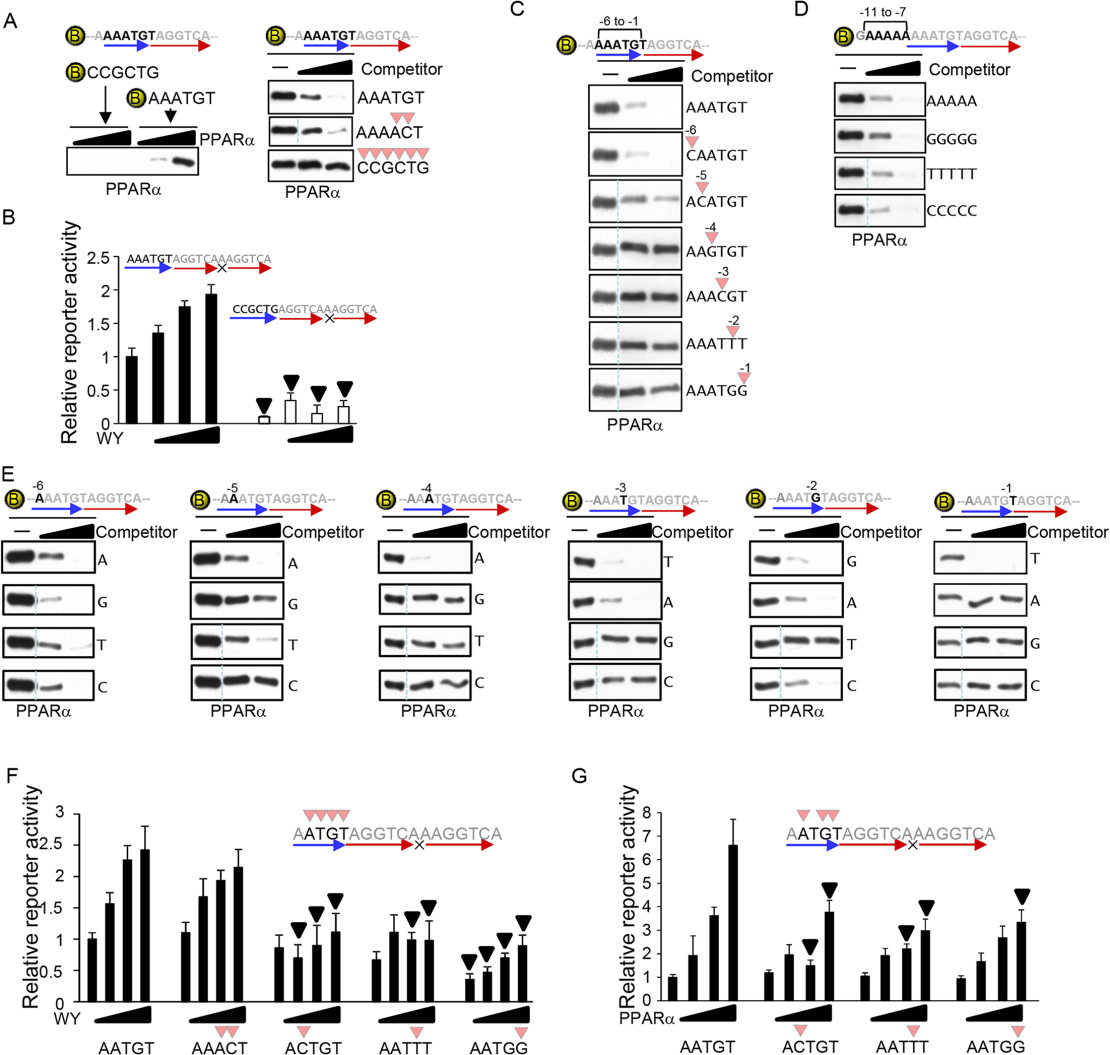
Determination of the 5’ extended sequence of the PPRE. (A) The DNA sequence of the 5’ extended region of DR1 is critical for PPARα binding. Biotin-labeled DNAs comprised of DR1 with the most and least frequent sequences in the 5’ extended region shown in Fig 1G were incubated with recombinant PPARα. PPARα bound to DNA was detected by Western blot analyses. (B) The 5’ extended sequence of DR1 is critical for PPARα ligand-induced transcriptional activation. Luciferase reporter genes driven by the PPREs/DR1s with the most and least frequent 6-bp sequences of the 5’extended region were stimulated with WY14643. (C) PPARα recognizes 5 bp of the 5’ extended region of the 5’ core hexad element. The competitors have less frequent nucleotides from positions -6 to -1. (D) PPARα does not recognize the 5’ extended region from position -7. The competitors have the indicated DNA sequences from -7 to -11. (E) Determination of the optimized PPARα binding sequence in the 5’ extended region. The competitors have all 4 possible nucleotides from positions -6 to -1. (C-E) The competition assays of PPARα binding were performed with biotin-labeled DNA and unlabeled competitors (10- and 30-fold excess of biotin-labeled DNA). (C- E) All signals without competitors are identical among the 4 panels at each position. A dotted line indicates discontinued signal but they are derived from identical blot/membrane. (F-G) The effect of a non-preferred nucleotide in the 5’ extended region on PPARα-induced transcriptional activation. Reporter gene assays were performed with the indicated PPRE sequences (n = 5–9).

In addition to the core hexad element, there was a sequence trend in approximately 6 bp of the 5’ extended region of the PPARα binding hexad element in the heart (Fig 1H). The most frequent nucleotide sequence is represented by AAATGT. The sequence of the 5’ extended region is reported to have an effect on the DNA binding of PPARα as a PPARα/RXRα dimer entity [15,16]. To investigate whether the 5’ extended sequence affects the binding of PPARα as a monomeric unit, DNA pull-down assays were performed using a 6-bp sequence that demonstrated the lowest frequency in the 5’ extended region (CCGCTG). Biotin-labeled PPARα binding sequence possessing the AAATGT extension was significantly pulled down with recombinant PPARα, but the sequence possessing the CCGCTG extension was not (Fig 4A, left). The consensus sequence of the 5’ extended region determined by this study in the heart (AAATGT) was slightly different from the one previously reported (AAAACT) [15]. However, we confirmed that both sequences bound equally to PPARα as a monomeric unit (Fig 4A, right). PPARα ligand-induced transcriptional activation was also significantly reduced when the perfect PPRE harbored the CCGCTG 5’ extended sequence as compared to AAATGT (Fig 4B). Thus, the 5’ extended sequence contributes to both PPARα binding and transcriptional activation.

To determine which nucleotide positions contribute to PPARα binding in the 5’ extended region, competition assays were performed with double-stranded DNA containing the lowest frequency nucleotide at each position. As shown in Fig 4C, the lowest frequency nucleotide at position -6 did not affect PPARα binding, while those from -5 to -1 significantly reduced PPARα binding, suggesting that PPARα recognizes 5 bp of the 5’ extended region. To test whether a 5’ nucleotide sequence far from -6 position affects PPARα DNA binding, repeated single nucleotides were introduced at positions -11 to -7. PPARα equally bound to all four kinds of repeated nucleotide sequences (Fig 4D). The results suggest that PPARα recognizes 5 bp of the 5’ extended region but not positions far from -7.

To determine the optimal PPARα binding sequence in the 5’ extended region, competition assays were performed at positions -6 to -1. As shown in Fig 4E, nucleotides positioned at -6 did not significantly differ in their effects on PPARα binding. However, PPARα clearly demonstrated binding preferences at positions -5 to -1, with WAWVT (W: A and T; V: A, G, and C) being the optimal sequence in the 5’ extended region for PPARα binding (Fig 4E and S2 Fig). The effect of nucleotide substitution within the 5’ extended region was examined by reporter gene assays. We selected nucleotide substitutions of A to C at the -4 position, G to T at -2, T to G at -1, and TG to AC at -3 to -2. The TG to AC nucleotide substitution did not affect PPARα binding and had no effect on PPARα-induced transcription, whereas the other substitutions reduced PPARα binding and attenuated PPARα ligand- and overexpression-induced transcriptional activation (Fig 4F and 4G). Taken altogether, these results indicate that PPARα recognizes a 5-bp segment in the 5’ extended region of the core hexad element with an optimal sequence of WAWVT, which contributes to PPARα binding and transcriptional activation.

### The optimized 5’ core hexad sequence of the PPRE

**Fig 5:**
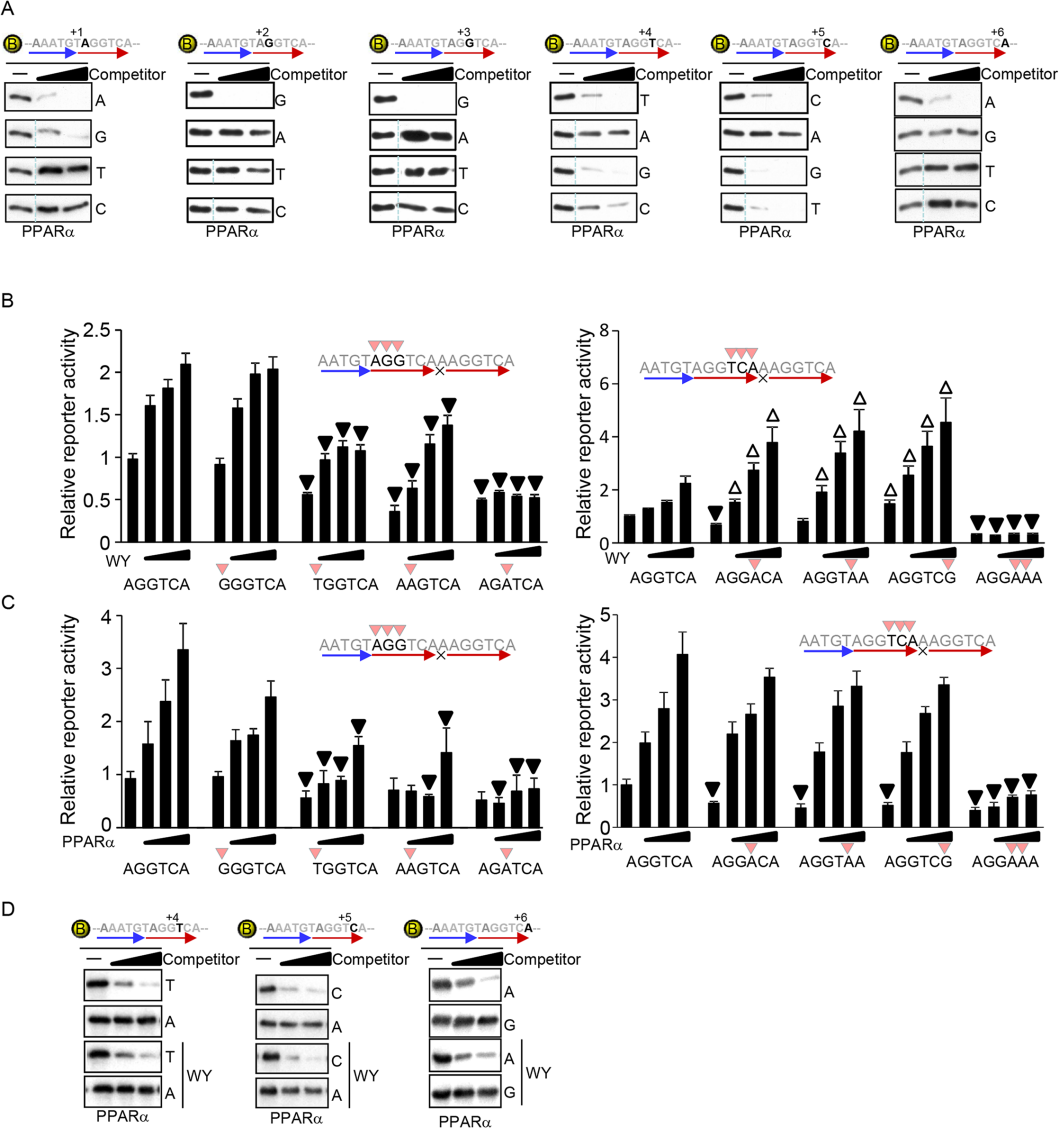
The 5’ core hexad sequence for PPARα binding. (A) Determination of the optimal PPARα binding sequence in the 5’ core hexad sequence. The competitors have all four possible nucleotides from positions +1 to +6. The competition assays of PPARα binding were performed with biotin-labeled DNA and unlabeled competitors (10- and 30-fold excess of biotin-labeled DNA). All signals without competitors are identical among the four panels at each position. A dotted line indicates a discontinued signal but they are derived from identical blots/membranes. (B-C) The effect of non-preferred nucleotides in the 5’ core hexad sequence on PPARα-induced transcriptional activation. Reporter gene assays were performed with the indicated PPRE sequences (n = 6–12). (D) PPARα ligand does not change PPARα binding sequence of DNA. The biotin-labeled DNA pulldown assays were performed with indicated competitors in the presence of 1 μM WY14,643.

The same strategy used to determine the PPARα binding sequence in the 5’ extended region was applied to determine the 5’ core hexad element. These sequential binding assays revealed that RGGBBA (R: A and G, B: G, T and C) is the optimal core hexad sequence to which PPARα preferentially binds (Fig 5A and S3 Fig).

To examine the effect of nucleotide substitution within the core element, reporter assays were performed. The presence of either nucleotide A or G at position +1 is an allowed redundancy for PPARα binding and both sequences were equally activated by the PPARα ligand, whereas T at the same position reduced PPARα binding significantly and attenuated the activation (Fig 5B). Nucleotide substitution to A from G nucleotides at positions +2 or +3 also reduced reporter activity induced by the PPARα ligand (Fig 5C). These results suggest that nucleotide substitutions that reduce PPARα binding in the region from +1 to +3 attenuate PPARα-induced transcription. In sharp contrast, single nucleotide substitutions that reduce PPARα binding at positions +4 to +6 did not significantly affect PPARα overexpression-induced reporter activity, while these substitutions unexpectedly enhanced ligand-induced reporter activity (Fig 5B). The enhanced transcriptional activation may be because the PPARα-preferred binding sequence of DNA is changed in presence of the ligand. However, the two nucleotide substitutions of AA for TC at positions +4 to +5 significantly impaired PPARα overexpression- and ligand-induced transcriptional activation (Fig 5B and 5C). Furthermore, *in vitro* DNA binding assays revealed that the DNA binding of PPARα was inhibited by nucleotide substitutions of T to A at position +4, C to A at position +5, and A to G at position +6 regardless of the presence of the ligand (Fig 5D). These results suggest that the PPARα-preferred binding sequence of DNA is not changed in the presence of the ligand. Taken altogether, a moderate attenuation of PPARα DNA binding caused by a single nucleotide substitution, but not by substitution of two nucleotides, at positions +4 to +6 of the hexad element enhances PPARα ligand-induced transcription.

### The optimized 3’ core hexad sequence of the PPRE

**Fig 6:**
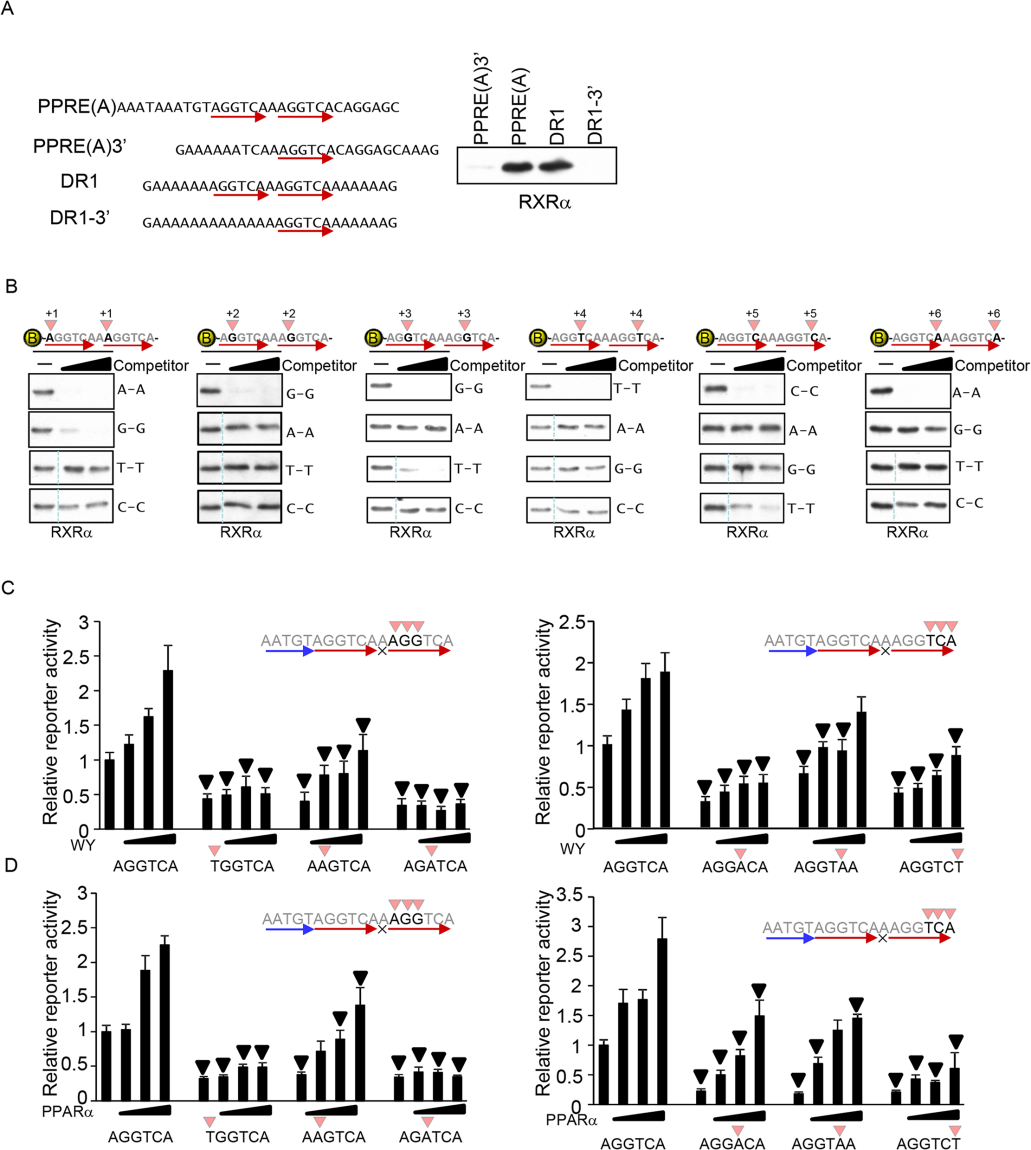
The 3’ core hexad sequence for RXRα binding. (A) Tandem AGGTCA sequences are required for detecting RXRα DNA binding. The indicated biotin-labeled DNA was incubated with recombinant RXRα. (B) Determination of the optimal DNA sequences for RXRα binding. Biotin-labeled double-stranded oligonucleotides comprising DR1 shown in Fig 6A were incubated with recombinant RXRα and unlabeled competitors (10- and 30-fold excess of biotin-labeled DNA). The competitors had all 4 possible nucleotides at the indicated positions (+1 to +6). All signals without competitors are identical among the four panels at each position. A dotted line indicates a discontinued signal but they are derived from identical blots/membranes. (C-D) The effect of non-preferred nucleotides in the 3’ core hexad sequence for RXRα binding on PPARα-induced transcriptional activation. Reporter gene assays were performed with the indicated sequences of PPRE (n = 6–9).

RXR binds to the 3’ core hexad sequence of the PPRE. To identify the optimal 3’ core hexad sequence, we performed DNA pull-down assays using recombinant RXRα and biotin-labeled DNA. As shown in Fig 6A, biotin-labeled DNA that included the sequence identified as the putative PPRE in the heart (Fig 1H) but that did not contain the PPARα binding half-site (PPRE(A)3’) was used first. RXRα did not significantly bind to the PPRE(A)3’ containing only the single AGGTCA sequence, although RXRα did significantly bind to PPRE(A), which is composed of two AGGTCA sequences. The proximal sequence of the RXR binding site of the core hexad element of the putative PPRE (Fig 1H) may coincidently comprise a certain sequence that reduces RXRα DNA binding. However, the same result was observed when the perfect DR1 and a single AGGTCA sequence were introduced into a sequential A sequence. These results suggest that binding of RXRα to DNA is highly dependent on dimer formation, despite the fact that monomeric PPARα strongly binds to DNA under the same experimental conditions.

To determine the optimal RXRα binding sequence, we used biotin-labeled DNA comprising the perfect DR1 sequence on the sequential A backbone (DR1). Nucleotide substitutions were concomitantly introduced on both sides of the hexad element in the unlabeled competitors. The DNA binding assay revealed that RGKTYA (R: A and G; K: G and T; Y: T and C) is an optimized RXRα binding sequence (Fig 6B and S4 Fig). Reporter gene assays were then performed to examine the effect of nucleotide substitution within the 3’ core element. Both PPARα ligand- and PPARα-overexpression-induced reporter gene activation were reduced by nucleotide substitutions that reduce RXRα DNA binding at all positions in the 3’ core element. These results suggest that stronger binding of RXR to the PPRE leads to more effective transcriptional activation.

### The PPRE sequence differentially directs transcriptional activation by liganded and unliganded PPARα

**Fig 7:**
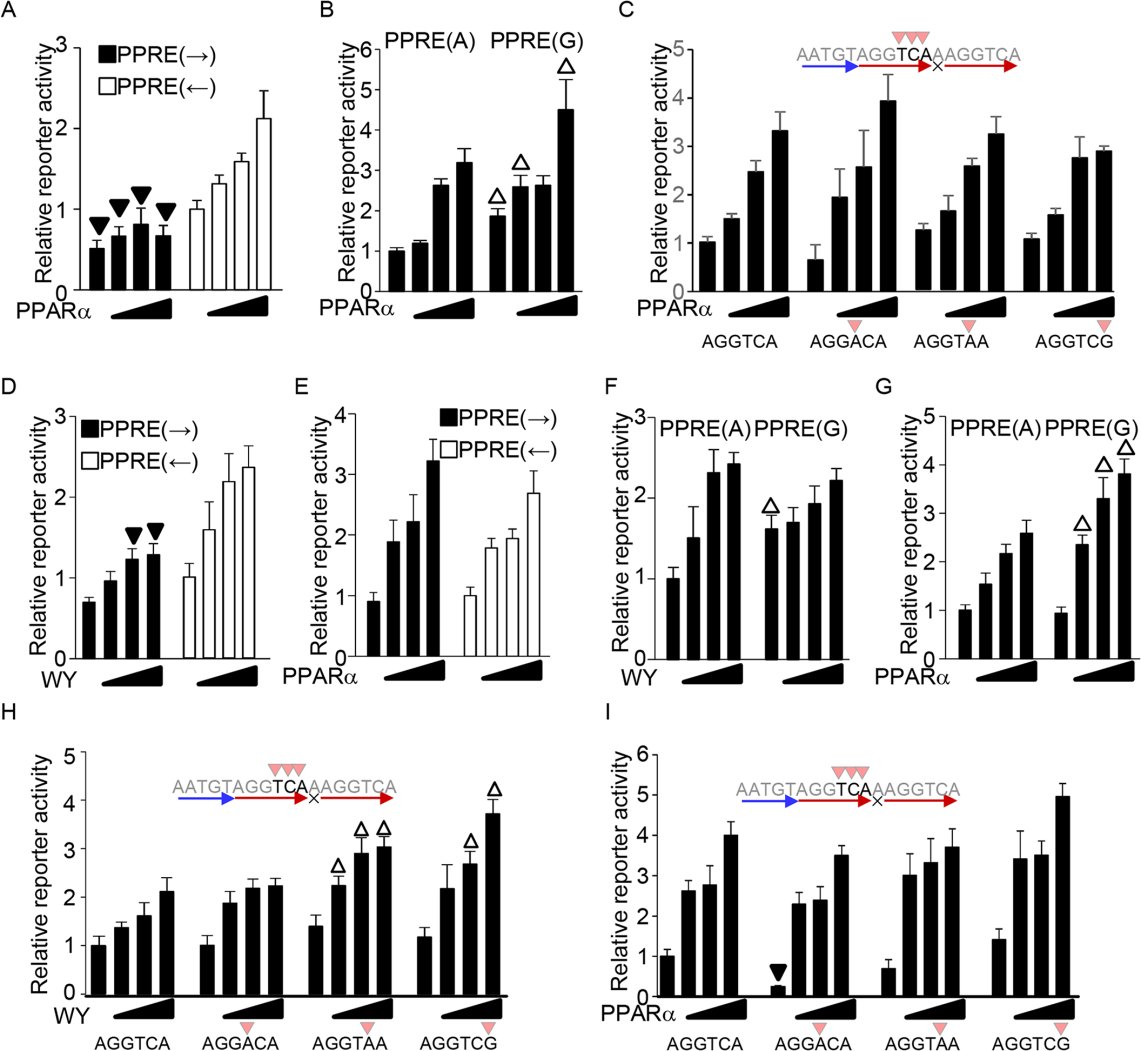
PPRE sequence differentially directs transcriptional activation by liganded and unliganded PPARα. (A) Forced expression of PPARα preferentially activates the major direction of the PPRE in primary cultured myocytes. (B) Overexpression of PPARα-induced transcriptional activation was enhanced with the PPRE containing a G nucleotide spacer in primary cultured myocytes. (C) Single nucleotide substitutions that reduce PPARα binding at positions +4 to +6 did not significantly affect PPARα-overexpression-induced reporter activity in primary cultured myocytes. (D-E) The effect of PPRE orientation in the ligand (D) and PPARα-overexpression (E)-induced transcriptional activation in Cos7 cells. (F-G) The effect of G nucleotide spacer in the ligand- (F) and PPARα-overexpression (G)-induced transcriptional activation in Cos7 cells. (H-I) The effect of nucleotide substitution that reduced PPARα DNA binding in the ligand (H) and PPARα-overexpression (I)-induced transcriptional activation in Cos7 cells. Reporter gene assays were performed with the indicated sequences of PPRE (n = 5–6).

Our results unexpectedly demonstrate that some PPRE sequences are not identically regulated by the PPARα ligand and by forced expression of PPARα such as orientation of the PPRE (Fig 2), G nucleotide spacer (Fig 3), and the last 3 nucleotide positions in 5’ hexad sequences (Fig 5). These results suggest that the PPRE sequence differentially directs transcriptional activation by liganded and unliganded PPARα. However, the differential transcriptional outcomes by the ligand and by PPARα overexpression may be due to differential cell types between primary and immortalized H9c2 myocytes. In addition, the serum in culture medium for H9c2 may contain fatty acid ligands for PPARα, which makes obscure whether the PPARα-overexpression-induced transcriptional activation is mediated by unliganded PPARα. To further test whether the PPRE sequence differentially directs transcriptional activation by liganded and unliganded PPARα, reporter gene assays were performed in primary cultured myocytes with PPARα overexpression. Notably, the primary cultured myocytes were cultured in the absence of serum after transfection of reporter and PPARα expression vectors. As shown in Fig 7A–7C, the effects of the PPRE sequence in PPARα-overexpression-induced transcriptional activation in primary cultured myocytes were identical to those in H9c2 cells. Overexpression of PPARα more strongly activated transcription when the orientation of the sequential PPARα and RXR binding site was opposite that of the luciferase gene body in primary cultured myocytes (Fig 7A). PPARα-overexpression-induced transcriptional activation was enhanced with the PPRE containing a G nucleotide spacer in primary cultured myocytes (Fig 7B). Single nucleotide substitutions that reduce PPARα binding at positions +4 to +6 did not significantly affect PPARα-overexpression-induced reporter activity in primary cultured myocytes (Fig 7C). These results suggest that the PPRE sequence differentially directs transcriptional activation by liganded and unliganded PPARα.

To investigate whether the transcriptional regulatory role of the PPRE sequence is specific for cardiac myocyte lineages, reporter gene assays were performed in Cos7 fibroblast-like cells. For the ligand treatment in Cos7 cells, a PPARα expression vector was co-transfected with the reporter genes, because the immortalized cell line does not express PPARα. The PPARα ligand more strongly activated transcription when the orientation of the sequential PPARα and RXR binding site was opposite that of the luciferase gene body in Cos7 cells (Fig 7D), whereas overexpression of PPARα equally activates transcription in both directions (Fig 7E). Thus, orientation of the PPRE has a regulatory role in PPARα-induced transcriptional activation in Cos7 cells, but the substantive regulation is different from that in myocyte cell lineages. Except for the orientation of the PPRE, the effects of the PPRE sequence in PPARα ligand- and overexpression-induced transcriptional activation in Cos7 cells were similar to those in myocytes. Transcriptional activation induced by the PPARα ligand was not significantly changed with the PPRE containing a G nucleotide spacer (Fig 7F), whereas that by PPARα overexpression was enhanced (Fig 7G). As is different from myocytes, single nucleotide substitutions at positions +4 in the 5’ hexad sequence did not significantly affect ligand-induced reporter activity, whereas those at positions +5 and +6 enhanced ligand-induced reporter activity in Cos7 cells (Fig 7H). Consistent with myocytes, these substitutions at positions from +4 to +6 did not significantly affect PPARα-overexpression-induced reporter activity in Cos7 cells (Fig 7I). These results suggest that the transcriptional regulatory behavior mediated by the PPRE sequence is not specific for cardiac myocyte lineages, except for the orientation of the PPRE.

### Identification of a novel PPARα response element

**Fig 8:**
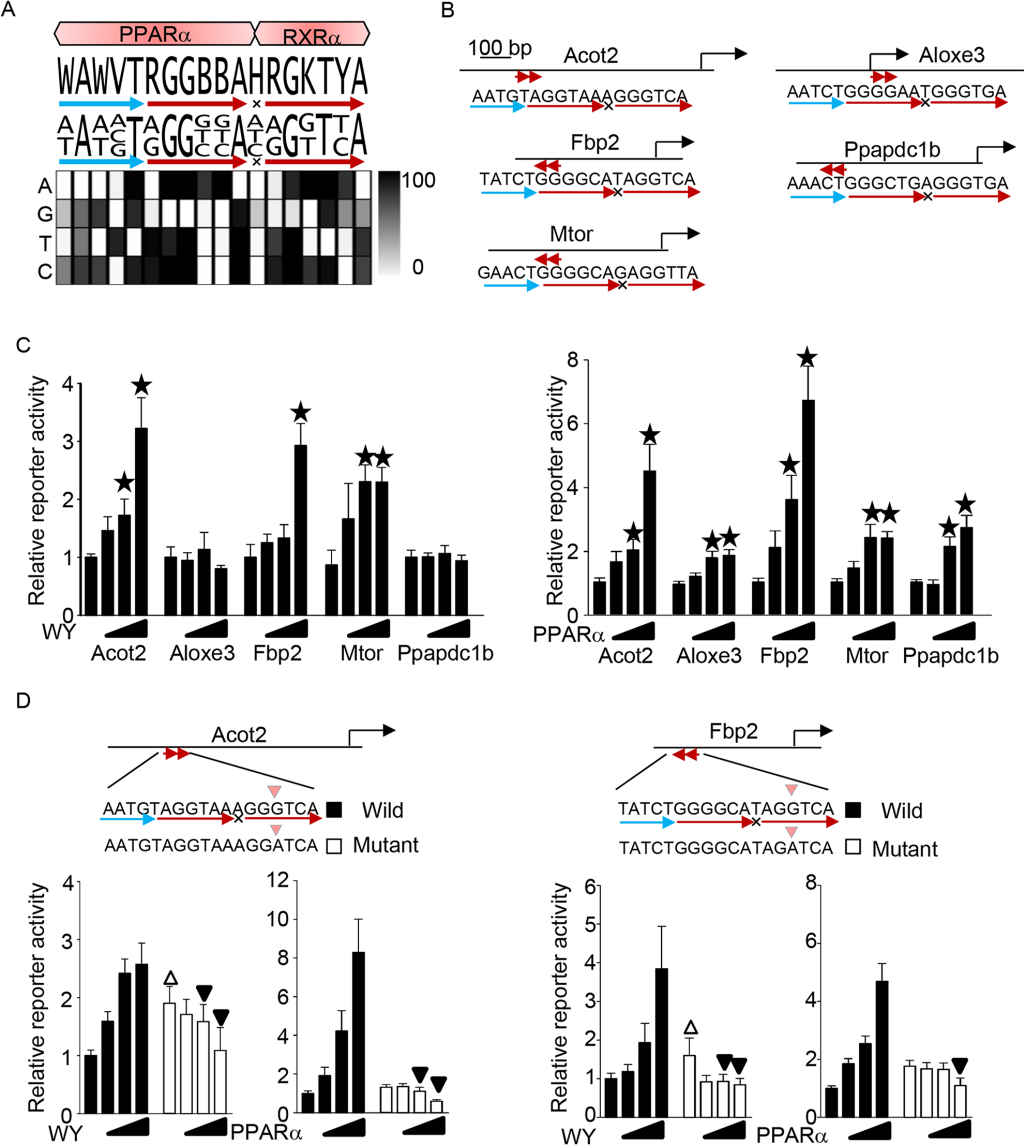
Identification of novel PPARα response elements using the determined sequence definition. (A) The optimized binding sequence for PPARα and RXRα (top). The densitometric analysis of the Western blot analyses (Figs 3 to 6) is shown by heat map (bottom). Signal densities were measured with the ImageJ program. The signal derived from a non-competitor was defined as 100. The optimized binding sequence was defined by the nucleotide sequences representing more than 80% competitive inhibition of the PPRE sequence we used (AAATGT-AGGTCA-A-AGGTCA). (B) Schematic representation of the reporter constructs driven by the promoters of the indicated genes. The potential PPRE is indicated by red arrows. (C) Reporter gene activation by PPARα. (D) Single nucleotide substitution at the +3 position of the 3’ hexad element of the putative PPRE in the *Acot2* and *Fbp2* promoters abolished PPARα-induced promoter activation. (C-D) The indicated reporter gene constructs were transfected into cells. Reporter assays were performed (n = 5–18).

Fig 8A shows a summary of the PPARα response element identified by this study. The optimal PPARα and RXRα binding sequences were WAWVT-RGGBBA-H and RGKTYA, respectively, making the optimal overall PPARα/RXR binding sequence WAWVT-RGGBBA-H-RGKTYA. As shown in the heat map, a 30-fold excess amount of this sequence competitively inhibited the PPRE sequence we used (AAATGT-AGGTCA-A-AGGTCA) by more than 80%. Several other sequences bound only weakly to PPARα or RXRα compared to the optimized sequences (Fig 8A). If these sequences are allowed as acceptable redundancies, the PPRE sequence becomes DAWVT-RGGBBA-N-RGKTBA, representing 60–80%, or AWVT-RGGBBA-N-RGKTBR, representing 50–60% competitive inhibition. Single nucleotide substitutions in positions +4 to +6 that reduced binding of PPARα to the PPARα binding hexad element and inclusion of a spacer position did not attenuate transcriptional activation, but rather enhanced transcription induced by a ligand or PPARα overexpression. Thus, stronger binding of PPARα to the DNA does not always lead to more effective transcriptional activation. To examine whether the improved definition of the PPRE is useful for identifying endogenous PPREs, we searched the promoter regions (± 2 kb from transcription start site) of the mouse genome for the PPRE sequence (DAWVT-RGGBBA-N-RGKTBA) that represented more than 60% competitive inhibition, allowing 1 mismatch in the last 3 bp of the 5’ hexad element. The bioinformatics analysis identified 176 potential PPREs (S1 Table). To evaluate if these PPREs are functional, we chose 5 intrinsic promoters, namely *Acot2*, *Aloxe3*, *Fbp2*, *mTOR*, and *Ppapdc1*, and generated luciferase reporters driven by them (Fig 8B). As shown in Fig 8C, the PPARα ligand stimulated the promoter activities of *Acot2*, *Fbp2*, and *mTOR*, but not of *Aloxe3* and *Ppapdc1b*. On the other hand, overexpression of PPARα was able to stimulate all the promoters. These results suggest that the improved definition of the PPRE is useful for identifying endogenous PPREs for PPARα, with the limitation that PPARα ligand-induced transcriptional activation is not observed in some of the identified promoters. To examine whether the identified PPRE is a major site for PPARα-induced activation, single nucleotide mutations from G to A at the +3 position of the RXR binding hexad element were generated in the *Acot2* and *Fbp2* promoters, because this mutation terminates PPARα-induced reporter gene activation (Fig 6C and 6D). This single nucleotide substitution in approximately 500 bp of the promoter abolished PPARα ligand- and PPARα-overexpression-induced reporter gene activation (Fig 8D). These results demonstrate that PPARα stimulates reporter gene activity through the PPRE identified.

## Discussion

Because of their pathophysiological significance in fatty acid metabolism, PPARs are among the most extensively investigated nuclear receptor subfamilies. Despite the fact that PPARs primarily function through sequence-specific DNA binding, the DNA sequences with which PPARs interact have not been fully characterized. Here we have shown that WAWVT-RGGBBA-H-RGKTYA is the optimal PPRE sequence for PPARα and RXRα binding. We have also shown the effect of nucleotide substitutions throughout the PPRE that reduce DNA binding of PPARα or RXR upon PPARα-induced transcription. The PPRE sequence determined in this study is present in endogenous gene promoters (S1 Table). Using the PPRE sequence, novel PPREs have been successfully identified, but these PPREs need more verification with alternative approaches such as chromatin immunoprecipitation analyses. Although there are several limitations, this study is currently the most comprehensive characterization of the PPRE sequence for PPARα.

Although the PPARα/RXR heterodimer is a functional entity for transcriptional activation, we determined the optimized binding sequence of monomeric PPARα for 3 reasons. First, we have shown that monomeric PPARα functionally suppresses transcription through DNA binding[8]. An isolated AGGTCA-like sequence is a major binding site for PPARs as evidenced by ChIP-on-Chip and ChIP-sequencing analyses [17–20]. Determination of a monomeric PPARα binding sequence is important in order to identify the target gene and elucidate the biological significance. Second, simplifying the experimental procedure is important for the data interpretation. Since PPARα and RXRα bind to similar AGGTCA-like sequences, reduced PPARα DNA binding is possibly caused by enhanced binding of the RXRα homodimer, if the binding reaction mixture contains both PPARα and RXRα and the oligonucleotide contains two AGGTCA-like sequences. Lastly, the monomeric PPARα preferred binding sequence may be changed when PPARα forms the heterodimer with RXR. To address this, the monomeric PPARα preferred binding sequence should be determined.

In this study, we searched for the PPRE only ± 2 kb from the transcription start site, because the comprehensive bioinformatics analyses are not a major aim of this study. The PPRE sequence used for the screening was relatively strict, because endogenous PPREs thus far identified have several nucleotide sequences that reduce the binding to PPARα and/or RXRα (Fig 1F vs Fig 8A). More PPREs should be found if longer promoter regions are searched using more redundant PPRE sequences. The PPRE sequence definition provided by this study should be an excellent resource for comprehensive bioinformatics analyses to identify the PPREs.

The biotin-labeled DNA pull-down assay is a well-established method for detecting sequence-specific DNA binding for a DNA binding protein. However, it has not previously been used to determine an optimized DNA sequence for nuclear receptor binding and we are the first to apply this method to determine the optimal DNA sequence for PPARα and RXRα binding. The major weakness of this method is the limitation in the ability to test a variety of DNA sequences. We tested all four nucleotides at single positions throughout the PPRE but were unable to test any combinational changes at several positions. A possible alternative method to address this limitation is systematic evolution of ligands by exponential enrichment (SELEX). In this method, a recombinant protein, such as a transcription factor, is incubated with random sequences of oligonucleotides and the oligonucleotide that binds to the protein is sequenced. Thus, SELEX is able to test all combinations of DNA sequences for transcription factor binding. However, there are several difficulties in applying the SELEX method to PPARs. Applying SELEX to a heterodimer transcription factor is generally challenging and requires optimal experimental conditions. Heterodimerization with RXR has generally been considered to be essential for PPARα DNA binding, although this and our previous studies have shown that monomeric PPARα is able to bind to DNA as well [8]. In addition, the RXR homodimer can bind to AGGTCA sequences with multiple configurations, such as DR1, DR0, and inverted repeat 0 (IR0). Therefore, it is necessary to isolate or distinguish the oligonucleotides that bind to the PPARα/RXR heterodimer but not to the RXR homodimer from the binding mixture containing both PPARα and RXR. Because RXR was thought to be essential for PPARα DNA binding, SELEX was performed for the PPARα/RXRα heterodimer[21]. However, the identified binding sequences contain DR0 to DR7, IR0, and more than two hexad elements, similar to the SELEX results obtained from analyzing the RXRα homodimer. Thus, optimizing the SELEX experimental conditions to elucidate the binding sequence for the PPARα/RXR heterodimer in the DR1 configuration seems to be challenging. Importantly, this study showed that there exist experimental conditions under which it is possible to detect sequence-specific DNA binding of monomeric PPARα and RXR homodimers. Thus, SELEX can be used to study monomeric PPARα and RXR homodimers separately, which could be a solution for the experimental difficulties encountered when applying SELEX to the PPARα/RXR heterodimer.

In addition to the experimental difficulties specific to PPARα/RXR, SELEX requires state-of-the-art technology and equipment, including preparation and verification of randomized double-stranded oligonucleotides, enrichment of the binding oligonucleotides, and a large scale sequencing and computational program for analyzing the data. In contrast, none of these state-of-the-art technological methods are necessary for the method we used in this study, making it accessible to research scientists across the globe. We expect that the biotin-labeled DNA pull-down assay can be feasibly applied to many nuclear receptors, contributing significantly to the understanding of their biological functions and molecular actions.

Several reports have provided clues regarding the sequence properties of the PPRE. The preferred binding sequence for the PPARα/RXRα heterodimer was found to be RGSWVA-N-AGGTCA (R = A or G; S = G or C; W = A or T; V = C, G, or A) by electrophoretic mobility shift assay (EMSA)[22]. These optimized core hexad sequences are similar but not completely identical to our result. It has also been reported that PPARα recognizes the 5’-extended region of the core hexad element [15,16]. However, the optimized PPARα binding sequence and the number of nucleotides PPARα recognizes in that region were unknown. Here we show that PPARα recognizes 5 bp of the 5’ extended region and that the optimized sequence is WAWVT. In an analysis of spacer nucleotides, it was found that a C spacer attenuates DNA binding of both PPARα/RXRα and PPARγ/RXRα [21,23]. We also demonstrated that a C spacer slightly attenuated PPARα/RXRα heterodimerization (Fig 3C) and PPARα-induced transcriptional activation (Fig 3F and 3G). Since monomeric PPARα strongly binds to the AGGTCA sequence in the presence of the C spacer, it is likely that RXRα DNA binding is inhibited by the C spacer, thereby attenuating the heterodimerization and transcriptional activation. The RXRα binding sequence has been suggested to be RGKTCA based on the alignment of known functional response elements [24,25]. Our data indicates that RXRα also binds to additional redundant sequences, including RGKTYA and RGKTBR. These sequences are likely to be acceptable redundancies because they are found in the 3’ hexad elements of actual PPREs such as in the promoters of *Ascl3* (AGGTCG), *Cpt1a* (AGGTTA), and *Slc25a20* (AGGTCG). DR1 is also the binding sequence for the RXR homodimer, called the RXR response element (RXRE). However, whether the DR1 sequence is a PPRE, RXRE, or dual element is not clearly defined. We found that PPARα and RXRα preferentially bound to different core hexad sequences (RGGBBA vs RGKTBR) (Figs 4 and 5) and that the A spacer was required for PPARα-independent RXRα DNA binding (Fig 3B). Based on these facts, PPRE and RXRE may be distinguished based on the DR1 sequence. If the 5’ hexad sequence represents the PPARα preferred binding sequence rather than that of RXRα, then the DR1 is likely a PPRE and not RXRE. In addition, the 5’ hexad sequence represents the preferred binding sequence for both PPARα and RXRα when A is the spacer, so the DR1 is potentially a dual response element acting as both the PPRE and RXRE. Thus, the optimized binding sequences for PPARα and RXRα provided here represent a significant improvement over past reports, without any obvious disagreement.

This study yielded two unexpected results. First, attenuating PPARα DNA binding at specific positions potentiated PPARα-induced transcriptional activation. Second, some PPRE sequences were not identically regulated by the PPARα ligand and by forced expression of PPARα. Stronger DNA binding of a transcription factor has generally been considered to result in more effective transcriptional activation. Indeed, nucleotide substitutions that reduced PPARα or RXR DNA binding attenuated PPARα-induced transcriptional activation in many positions. Interestingly, however, a single nucleotide substitution at positions +4 to +6 in the 5’ hexad element reduced PPARα DNA binding but enhanced PPARα ligand-induced transcriptional activation compared to the perfect DR1, while PPARα-overexpression-induced activation was not changed (Fig 5B and 5C). In addition, a G in the spacer position reduced PPARα DNA binding but enhanced PPARα-overexpression-induced transcriptional activation, while the ligand-induced activation was not significantly changed (Fig 3F and 3G). The orientation of the PPRE relative to the gene is another example of differential regulation by the ligand and by forced expression of PPARα (Figs 2B, 2C, 7A, 7D and 7E). These results suggest that liganded and unliganded PPARα differentially regulate target gene expression. Although the pathophysiological significance of and the mechanism responsible for this differential regulation are currently unknown, it may explain the diverse functional outcomes mediated by PPARα.

In summary, this study identified WAWVT-RGGBBA-H-RGKTYA as an optimized DNA sequence for PPARα and RXRα binding. Stronger DNA binding of RXR on the PPRE/DR1 leads to more effective transcription, but this is not always true for PPARα. Several PPRE sequences are differentially regulated by the PPARα ligand and by upregulation of PPARα. This improved PPRE sequence characterization should contribute to a better understanding of PPARα signaling by enabling identification of previously undetected functional PPREs.

## Supporting Information

S1 ARRIVE Checklist (DOCX)

S1 Fig Purity of the recombinant PPARα and RXRα. Three μg proteins were loaded and stained with Coomassie Brilliant Blue.
(TIF)

S2 Fig Densitometeric analysis of the DNA binding assays between PPARα and the 5’ extended region (Fig 4E). Signal densities of the Western Blot analyses were measured using the ImageJ program (n = 3–9). The triangle indicates statistical significance compared to the consensus sequence of each position (left of each position).
(TIF)

S3 Fig Densitometeric analysis of the DNA binding assays between PPARα and the 5’ core hexad sequence (Fig 5A). Signal: The triangle indicates statistical significance compared to the consensus sequence of each position (left of each position).
(TIF)

S4 Fig Densitometeric analysis of the DNA binding assays between RXRα and the 3’ core hexad sequence (Fig 6B). Signal densities of the Western Blot analyses were measured using the ImageJ program (n = 4–7). The triangle indicates statistical significance compared to the consensus sequence of each position (left of each position).
(TIF)

S1 Table Bioinformatics search for PPREs in mouse genome. Bioinformatics search for PPREs in mouse genome. In Region, ups and dns indicate upstream and downstream of TSS, respectively. In Direction, fowrd indicates that the motif matches with the direction of the gene body, while bckwd indicates that the motif matches the reverse complement. The genomic position is based on mm9.
(PDF)
