# Supplementary figures and images for: An Ideal PPAR Response Element Bound to and Activated by PPARα
Source: PLoS One. 2015 Aug 4;10(8):e0134996. doi: 10.1371/journal.pone.0134996 (PMC4524655; doi:10.1371/journal.pone.0134996)

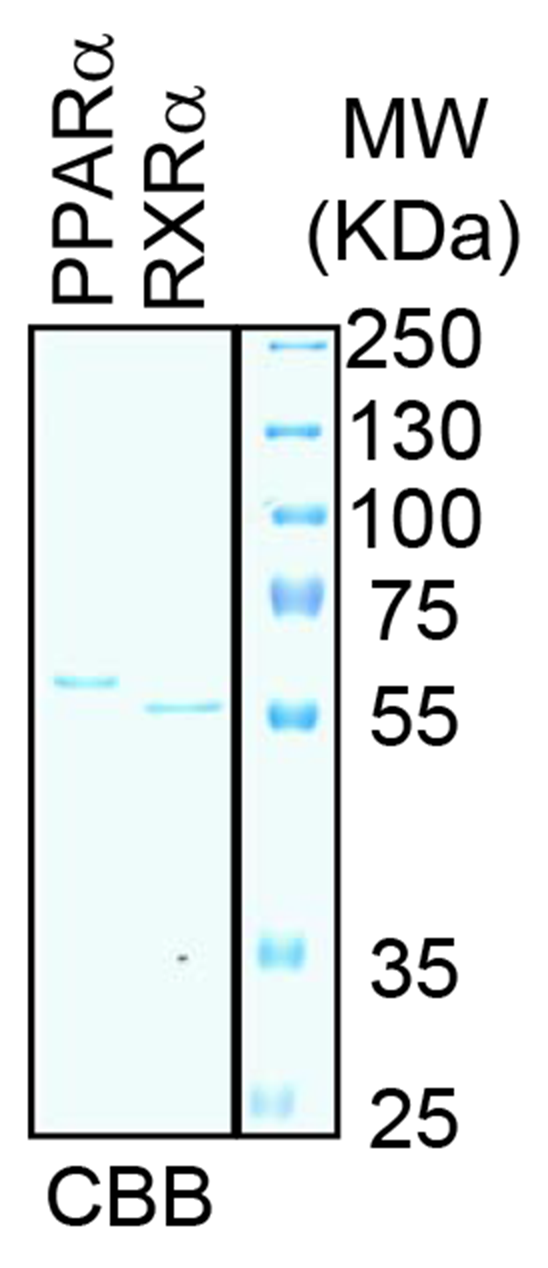

Supplement: S1 Fig — Three μg proteins were loaded and stained with Coomassie Brilliant Blue. (TIF) [file pone.0134996.s002.tif]

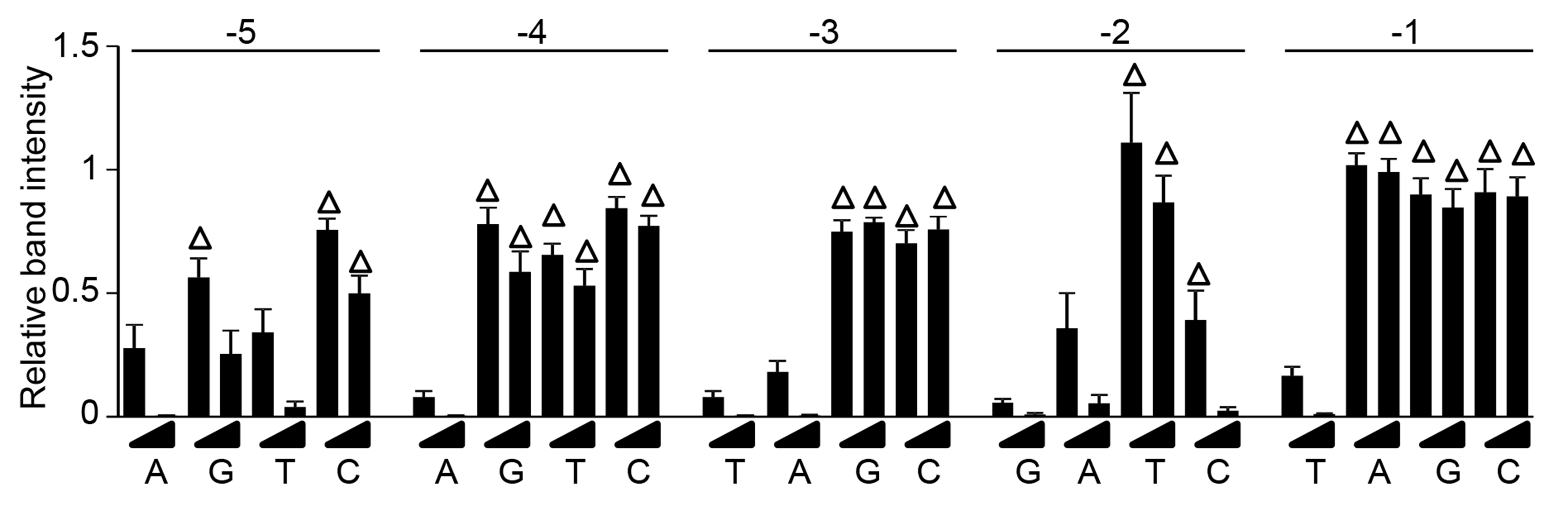

Supplement: S2 Fig — Signal densities of the Western Blot analyses were measured using the ImageJ program (n = 3–9). The triangle indicates statistical significance compared to the consensus sequence of each position (left of each position). (TIF) [file pone.0134996.s003.tif]

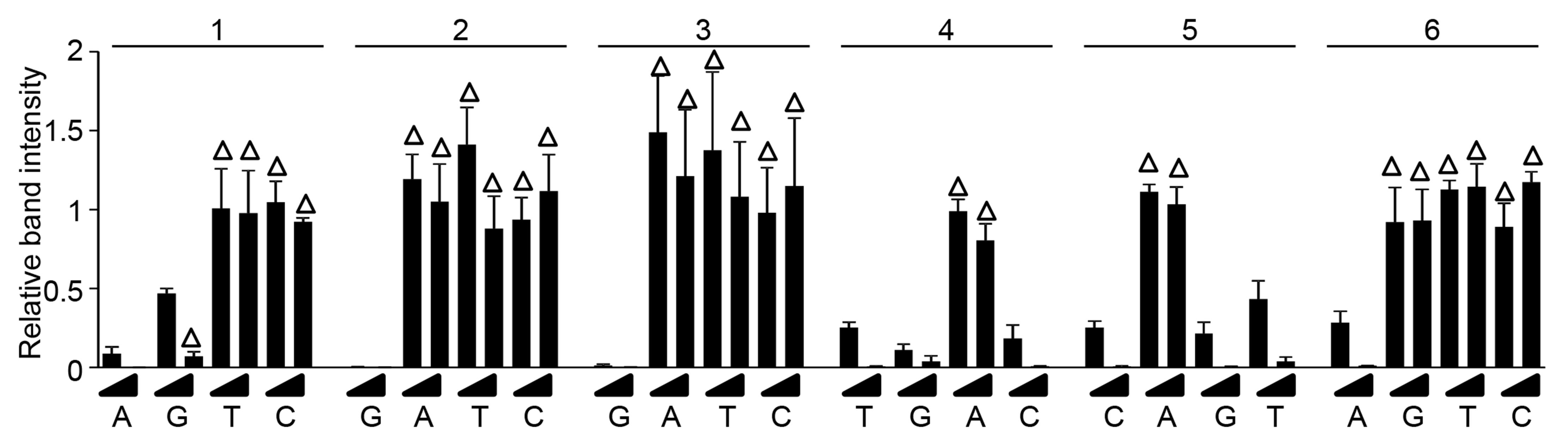

Supplement: S3 Fig — Signal: The triangle indicates statistical significance compared to the consensus sequence of each position (left of each position). (TIF) [file pone.0134996.s004.tif]

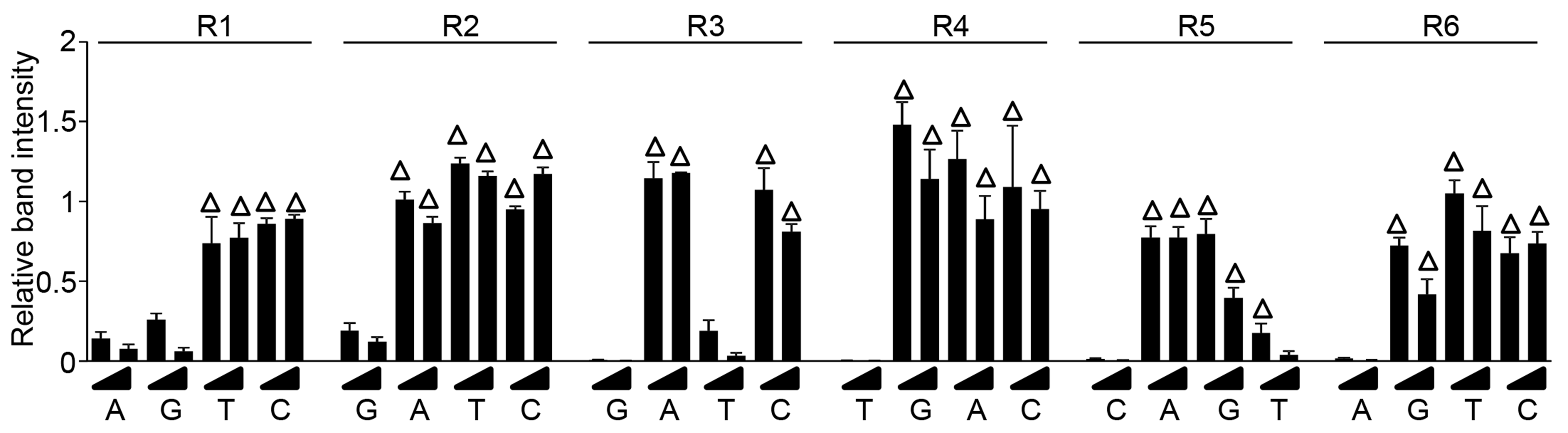

Supplement: S4 Fig — Signal densities of the Western Blot analyses were measured using the ImageJ program (n = 4–7). The triangle indicates statistical significance compared to the consensus sequence of each position (left of each position). (TIF) [file pone.0134996.s005.tif]
