# Supplementary material for: An Ideal PPAR Response Element Bound to and Activated by PPARα
Source: PLoS One. 2015 Aug 4;10(8):e0134996. doi: 10.1371/journal.pone.0134996 (PMC4524655; doi:10.1371/journal.pone.0134996)
Supplement: S1 Table — Bioinformatics search for PPREs in mouse genome. In Region, ups and dns indicate upstream and downstream of TSS, respectively. In Direction, fowrd indicates that the motif matches with the direction of the gene body, while bckwd indicates that the motif matches the reverse complement. The genomic position is based on mm9. (PDF) [file pone.0134996.s006.pdf]

Gene Name	Gene ID	Description	TSS	Promoter region	Motif	Upstream or downstream of TSS	Direction	Distance from TSS (bp)	Genomic position
1600015I10Rik	69761	RIKEN cDNA 1600015I10 gen	chr6:+:48879894	48877894:48881894	TATGTGGGTCAGAGTTCA	ups	bckwd	1102	48878791
2810007J24Rik	76971	RIKEN cDNA 2810007J24 gen	chr7:-:15023900	15021900:15025900	GATGTAGGTCAGGGGTTA	ups	forwd	77	15023978
4930404N11Rik	432479	RIKEN cDNA 4930404N11 ge	chr10:-:80828565	80826768:80830565	GATCTGGGGTCTGGGTGA	dns	bckwd	143	80828421
4930444G20Rik	114671	RIKEN cDNA 4930444G20 ge	chr10:-:21787885	21786114:21789885	AATATGGGTGAGGGGTTA	ups	bckwd	982	21788868
4930579G24Rik	75939	RIKEN cDNA 4930579G24 ge	chr3:+:79433000	79431000:79435000	AAACTAGGGCAAAGGTCA	ups	bckwd	679	79432320
9930012K11Rik	268759	RIKEN cDNA 9930012K11 ge	chr14:-:70559309	70557309:70561309	AATGTGGGTGATGGGTGA	dns	bckwd	332	70558976
AI182371	98870	expressed sequence AI1823	chr2:-:34956205	34954205:34958205	TAAGTAGGGTCAAGGTTA	dns	bckwd	1368	34954836
Aaed1	66129	AhpC/TSA antioxidant enzym	chr13:-:64414018	64412018:64416018	GAACTAGGCTTAAGTTTA	dns	bckwd	1141	64412876
Abi2	329165	abl-interactor 2	chr1:+:60466462	60464462:60468462	GAAATAGGGGAGGGTTTA	ups	bckwd	1720	60464741
Acaa1b	235674	acetyl-Coenzyme A acyltrans	chr9:-:119066211	119064211:119068211	TAAGTAGGGAAAAGGTTA	dns	bckwd	1489	119064721
Acot2	171210	acyl-CoA thioesterase 2	chr12:+:85328810	85326810:85330810	AATGTAGGTAAAGGGTCA	ups	forwd	460	85328349
Adgb	215772	androglobin	chr10:-:10192112	10190112:10194112	TAAATGGGGCTAGGGTGA	dns	bckwd	1506	10190605
Ago4	76850	argonaute RISC catalytic sub	chr4:-:126210702	126208702:126212702	TAAGTAGGGCAAGGTTTA	dns	forwd	1082	126209619
Aloxe3	23801	arachidonate lipoxygenase 3	chr11:+:68939878	68937878:68941878	AATCTGGGGAATGGGTGA	ups	forwd	406	68939471
Als2cr12	108812	amyotrophic lateral sclerosis	chr1:-:58752833	58750833:58754833	AATCTAGGGGATAGGTGA	dns	forwd	1600	58751232
Aqp11	66333	aquaporin 11	chr7:-:104886757	104884757:104888757	AAACTGGGGGAAAGGTTA	ups	forwd	1860	104888618
Armcx1	78248	armadillo repeat containing,	chrX:+:131252476	131250476:131254476	GAACTGGGGGCAGGGTGA	dns	forwd	867	131253344
Atp5e	67126	ATP synthase, H+ transportir	chr2:-:174289602	174287602:174291602	GATCTGGGTCCTAGGTGA	ups	bckwd	770	174290373
Bbs9	319845	Bardet-Biedl syndrome 9 (hu	chr9:+:22280158	22278158:22282158	TATATAGGTTATAGGTTA	ups	bckwd	424	22279733
Calhm2	72691	calcium homeostasis modula	chr19:-:47212784	47210784:47214784	GATCTGGGGTTAAGTTCA	ups	bckwd	976	47213761
Caln1	140904	calneuron 1	chr5:+:130845327	130843327:130847327	AATATGGGTTATAGGTTA	dns	forwd	770	130846098
Ccdc62	208908	coiled-coil domain containin	chr5:+:124380697	124378697:124382697	AAACTAGGGGGAAGGTTA	ups	bckwd	1394	124379302
Cda	72269	cytidine deaminase	chr4:-:137923870	137921870:137925870	AATATGGGTGAGGGGTCA	dns	forwd	1307	137922562
Cdk16	18555	cyclin-dependent kinase 16	chrX:+:20265618	20263618:20267618	TAAGTGGGAGAAGGGTGA	dns	forwd	543	20266162
Cenpi	102920	centromere protein I	chrX:+:130842701	130840701:130844701	AAACTGGGTGAGAGGTTA	ups	bckwd	527	130842173
Ciita	12265	class II transactivator	chr16:+:10480164	10478164:10482164	GAAATGGGTTAGGGGTGA	ups	bckwd	95	10480068
Cngb3	30952	cyclic nucleotide gated chan	chr4:+:19207996	19205996:19209996	AATCTGGGCCCTGGGTGA	ups	forwd	542	19207453
Coq5	52064	coenzyme Q5 homolog, metl	chr5:+:115729710	115727710:115731710	AATATAGGGTTAAGGTTA	dns	forwd	709	115730420
Cpt1b	12895	carnitine palmitoyltransferas	chr15:-:89256293	89254293:89258293	AATGTAGGGAAAAGGTCA	ups	bckwd	229	89256523
Cpt2	12896	carnitine palmitoyltransferas	chr4:-:107596194	107594194:107598194	TATGTAGGGGAAAGGTGA	dns	forwd	1495	107594698
Crx	12951	cone-rod homeobox	chr7:-:16457734	16455734:16459734	GAAATAGGCCTAAGTTGA	dns	forwd	544	16457189
D630033O11Rik	235302	RIKEN cDNA D630033O11 ge	chr9:+:43067961	43065961:43069961	GAAATAGGTAAAGGGTTA	ups	bckwd	1293	43066667
Ddx56	52513	DEAD (Asp-Glu-Ala-Asp) box	chr11:-:6167732	6165732:6169732	GAAATGGGCCTAAGGTCA	ups	forwd	965	6168698
Dennd6b	69440	DENN/MADD domain contai	chr15:-:89026905	89024905:89028905	GAAGTGGGGGAAAGGTCA	dns	bckwd	554	89026350
Dmtn	13829	dematin actin binding protei	chr14:-:71018376	71016376:71020376	GATGTAGGCCGAGGTTTA	ups	forwd	1747	71020124
Dnajb2	56812	DnaJ (Hsp40) homolog, subfa	chr1:+:75233276	75231276:75235276	GATGTGGGTGGTGGTTCA	dns	forwd	1803	75235080
Dusp9	75590	dual specificity phosphatase	chrX:+:70884779	70882779:70886779	TATATGGGGAAAGGTTGA	ups	bckwd	1132	70883646
Efcab6	77627	EF-hand calcium binding dor	chr15:-:83749803	83747803:83751803	GAAGTGGGCCGTGGGTCA	ups	forwd	567	83750371
Egfl7	353156	EGF-like domain 7	chr2:+:26436575	26434575:26438575	GAAGTAGGTGTTGGGTCA	ups	bckwd	1789	26434785
Etfdh	66841	electron transferring flavopr	chr3:-:79432689	79430689:79434689	AAACTAGGGCAAAGGTCA	dns	forwd	368	79432320
Fabp1	14080	fatty acid binding protein 1, l	chr6:+:71149881	71147881:71151881	GATATAGGCCATAGGTCA	dns	bckwd	57	71149823
Fam109b	338368	family with sequence similar	chr15:+:82171608	82169608:82173608	TAAGTGGGGGTAGGGTGA	ups	forwd	710	82170897
Fam43a	224093	family with sequence similar	chr16:+:30599808	30597808:30601808	GATATGGGCTCTAGTTTA	ups	forwd	1951	30597856
Fbp2	14120	fructose bisphosphatase 2	chr13:-:62959730	62957730:62961730	TATCTGGGGCATAGGTCA	ups	bckwd	383	62960114
Fbxo24	71176	F-box protein 24	chr5:-:138066306	138064306:138068306	TATATAGGGTTAGGGGTTA	ups	bckwd	1421	138067728
Fgfbp3	72514	fibroblast growth factor bind	chr19:-:36994089	36992089:36996089	AAAATGGGTCAGGGGTCA	dns	forwd	1262	36992826
Fgfrl1	116701	fibroblast growth factor rece	chr5:+:109123247	109121247:109125247	AATCTGGGGGAGGGGTGA	dns	bckwd	1260	109124508
Fhl1	14199	four and a half LIM domains	chrX:+:54032859	54030859:54034859	GAAATGGGCTTTGGTTGA	dns	forwd	1545	54034405
Fhl1	14199	four and a half LIM domains	chrX:+:54039703	54037703:54041703	AAAATAGGTGCTGGGTCA	ups	bckwd	1214	54038488
Galnt1	14423	UDP-N-acetyl-alpha-D-galact	chr18:+:24363844	24361844:24365844	AAAGTGGGGTGTAGGTGA	dns	bckwd	1403	24365248
Galnt1	14423	UDP-N-acetyl-alpha-D-galact	chr18:+:24364494	24362494:24366494	AAAGTGGGGTGTAGGTGA	dns	bckwd	753	24365248
Gcn1l1	231659	GCN1 general control of ami	chr5:+:116015271	116013271:116017271	TAACTGGGTCCTGGGTGA	dns	forwd	615	116015887
Ggta1	14594	glycoprotein galactosyltransf	chr2:-:35316945	35314945:35318945	AATATAGGTGAGGGGTTA	dns	forwd	1740	35315204
Gm10256	1E+08	predicted gene 10256	chrY:+:2156898	2154898:2158898	AATATAGGCAAAAGTTTA	dns	forwd	1375	2158274
Gm3376	1E+08	predicted gene 3376	chrY:+:2387257	2385257:2389257	AATATAGGCAAAAGTTTA	dns	forwd	1376	2388634
Gm609	208166	predicted gene 609	chr16:-:45493082	45491082:45495082	AAAATAGGTGTAAGGTTA	dns	bckwd	1719	45491362
Gmfg	63986	glia maturation factor, gamr	chr7:+:29225955	29223955:29227955	TATCTGGGAGATAGGTGA	ups	forwd	187	29225767
Grhl1	195733	grainyhead-like 1 (Drosophil	chr12:+:25257151	25255151:25259151	GATCTAGGGGAAGGTTGA	dns	forwd	412	25257564
Gsc2	195333	goosecoid homebox 2	chr16:-:17915152	17913646:17917152	AAAGTGGGAGATGGGTGA	ups	forwd	1299	17916452
Gtf2h4	14885	general transcription factor I	chr17:-:35810627	35808627:35812627	AATATAGGGCCTAGGTCA	ups	forwd	1589	35812217
H1f0	14958	H1 histone family, member C	chr15:+:78858641	78856641:78860641	GATGTGGGGAAAGGGTTA	ups	bckwd	1398	78857242
Hhatl	74770	hedgehog acyltransferase-lik	chr9:-:121701625	121699625:121703625	AATATGGGTGAGGGGTTA	ups	forwd	1621	121703247
Hic2	58180	hypermethylated in cancer 2	chr16:+:17233679	17231679:17235679	GATGTGGGTCAGGGGTGA	ups	forwd	29	17233649
Hilpda	69573	hypoxia inducible lipid dropl	chr6:+:29222487	29220487:29224487	AAAGTAGGGGAAAGGTCA	ups	forwd	1357	29221129
Hilpda	69573	hypoxia inducible lipid dropl	chr6:+:29222625	29220625:29224625	AAAGTAGGGGAAAGGTCA	ups	forwd	1495	29221129
Hist1h2bm	319186	histone cluster 1, H2bm	chr13:+:21813912	21811912:21814395	AAAGTAGGGGAAAGGGTGA	ups	bckwd	1331	21812580
Hist1h2bn	319187	histone cluster 1, H2bn	chr13:+:21845991	21843991:21846422	AAACTAGGGTTAGGTTCA	ups	forwd	815	21845175
Hpdl	242642	4-hydroxyphenylpyruvate dio	chr4:-:116494113	116492511:116496113	AATCTGGGTTAGAGGTGA	ups	forwd	1569	116495683
Hsd17b7	15490	hydroxysteroid (17-beta) deh	chr1:-:171899336	171897336:171901336	TAAGTAGGTCAAGGGTCA	dns	bckwd	576	171898759
Islr	26968	immunoglobulin superfamily	chr9:-:58006361	58004361:58008361	TAAATAGGGGAAGGGTGA	ups	bckwd	862	58007224
Islr	26968	immunoglobulin superfamily	chr9:-:58007028	58005028:58009028	TAAATAGGGGAAGGGTGA	ups	bckwd	195	58007224
Iyd	70337	iodotyrosine deiodinase	chr10:-:6806262	6804262:6808262	TAAATAGGAGAAAGGTCA	ups	forwd	305	6806568
Kcnab2	16498	potassium voltage-gated cha	chr4:-:151851658	151849658:151853658	TAAGTGGGGGATGGGTGA	dns	bckwd	1033	151850624
Kctd1	106931	potassium channel tetrameri	chr18:-:15309955	15307955:15311955	GAAGTAGGGGGAGGGTGA	dns	forwd	1396	15308558

Kdsr	70750	3-ketodihydrosphingosine re	chr1:-:108656319	108654319:108658319	AAACTAGGTGGAAGTTGA	ups	bckwd	1701	108658021
Kif2b	73470	kinesin family member 2B	chr11:-:91438869	91436869:91440869	TAAGTAGGTTAGAGTTTA	ups	bckwd	1635	91440505
Klf10	21847	Kruppel-like factor 10	chr15:-:38230466	38228466:38232466	GAAGTAGGGGAAAGGTGA	dns	forwd	1413	38229052
Klhl22	224023	kelch-like 22	chr16:+:17759713	17757713:17761713	GATATAGGTGATAGGTTA	ups	forwd	1757	17757955
Krt19	16669	keratin 19	chr11:-:100007233	100005233:100009233	GAAATAGGGTGTGGTTGA	ups	bckwd	1774	100009008
Ldlr	16835	low density lipoprotein recep	chr9:+:21528019	21526019:21530019	TATGTAGGCCAGAGTTCA	ups	bckwd	1960	21526058
Lhx3	16871	LIM homeobox protein 3	chr2:-:26062095	26060095:26064095	GAAATGGGTGCTGGGTGA	ups	bckwd	1077	26063173
Lhx3	16871	LIM homeobox protein 3	chr2:-:26063769	26061769:26065769	GAAATGGGTGCTGGGTGA	dns	bckwd	578	26063190
Lmna	16905	lamin A	chr3:-:88297234	88295234:88299234	AATATGGGTCTAGGTTGA	ups	forwd	111	88297346
Lonrf2	381338	LON peptidase N-terminal dc	chr1:-:38878060	38876060:38880060	AATGTGGGCGAGGGGTTA	ups	forwd	1414	38879475
Lrrc43	381741	leucine rich repeat containin	chr5:+:123939333	123937333:123941333	TATATAGGTGCTGGGTCA	dns	forwd	1269	123940603
Megf11	214058	multiple EGF-like-domains 1:	chr9:+:64233432	64231432:64235432	GAAATGGGCTTTGGTTTA	ups	forwd	1660	64231771
Mif	17319	macrophage migration inhibi	chr10:-:75322995	75322097:75324995	TATCTGGGCTCTGGTTCA	ups	bckwd	1589	75324585
Mmd	67468	monocyte to macrophage dif	chr11:+:90110789	90108789:90112789	TAAGTGGGTGAAAGTTCA	dns	forwd	1022	90111812
Ms4a4d	66607	membrane-spanning 4-doma	chr19:+:11611338	11609338:11613338	GAACTAGGTCAGAGTTTA	dns	bckwd	317	11611656
Mtap7d3	320923	MAP7 domain containing 3	chrX:-:54075502	54073502:54077502	TAACTAGGTCATAGTTTA	dns	forwd	1534	54073967
Mtor	56717	mechanistic target of rapamy	chr4:+:147822690	147820690:147824690	GAACTGGGGCAGAGGTTA	ups	bckwd	316	147822373
Myo1b	17912	myosin IB	chr1:-:51972822	51970822:51974822	TAAATGGGGCCAGGTTTA	ups	bckwd	658	51973481
Myo3a	667663	myosin IIIA	chr2:+:22149129	22147129:22151129	TATGTAGGTTTTGGTTTA	dns	forwd	1418	22150548
Naga	17939	N-acetyl galactosaminidase,	chr15:-:82169256	82167256:82171256	TAAGTGGGGGTAGGGTGA	ups	bckwd	1623	82170880
Nckap1l	105855	NCK associated protein 1 like	chr15:+:103284255	103282255:103286255	AATATGGGTGAGGGGTTA	ups	bckwd	1658	103282596
Nipal3	74552	NIPA-like domain containing	chr4:-:135050419	135048419:135052419	GAACTGGGCGGTGGGTGA	ups	bckwd	1756	135052176
Nphp4	260305	nephronophthisis 4 (juvenile	chr4:+:151852250	151850250:151854250	TAAGTGGGGGATGGGTGA	ups	forwd	1625	151850624
Nr2f2	11819	nuclear receptor subfamily 2	chr7:-:77505479	77503479:77507479	AATGTAGGGGGAGGGTGA	dns	bckwd	1900	77503578
Nsfl1c	386649	NSFL1 (p97) cofactor (p47)	chr2:+:151319917	151317917:151321917	GAAGTGGGGTCAGGTTCA	dns	bckwd	1979	151321897
Nt5c	50773	5',3'-nucleotidase, cytosolic	chr11:-:115353128	115351739:115355128	AAAATGGGGGCTGGGTGA	dns	forwd	713	115352414
Oacyl	319888	O-acyltransferase like	chr18:+:65857921	65855921:65859921	GAAATAGGGCAAAGGTTA	dns	bckwd	407	65857513
Obp2a	227627	odorant binding protein 2A	chr2:+:25555593	25553593:25557593	GAAGTGGGTCAGGGGTCA	ups	forwd	568	25555024
Obp2b	383678	odorant binding protein 2B	chr2:+:25592528	25590528:25594528	GAAGTGGGTCAGGGGTCA	ups	forwd	537	25591990
Olfr1044	259013	olfactory receptor 1044	chr2:-:86011972	86011027:86013972	TAACTGGGGGCAGGGTCA	ups	bckwd	1498	86013471
Olfr1261	258466	olfactory receptor 1261	chr2:+:89833551	89831551:89834472	GATCTGGGTCTTGGGTCA	dns	bckwd	34	89833586
Olfr1262	258976	olfactory receptor 1262	chr2:+:89842564	89840564:89843479	GATCTGGGTCTTGGGTCA	dns	bckwd	34	89842599
Olfr1263	258790	olfactory receptor 1263	chr2:+:89855088	89853088:89856009	GATCTGGGTCTTGGGTCA	dns	bckwd	34	89855123
Olfr1504	258627	olfactory receptor 1504	chr19:-:13962698	13961750:13964698	GATATAGGCTCTGGGTTA	ups	forwd	1415	13964114
Olfr1507	57269	olfactory receptor 1507	chr14:-:53115370	53113370:53117370	TAACTAGGCTCAAGGTCA	dns	bckwd	1874	53113495
Olfr316	258064	olfactory receptor 316	chr11:+:58571168	58569168:58572089	AAAATGGGGAAAAGGTGA	ups	forwd	1545	58569622
Olfr597	258135	olfactory receptor 597	chr7:+:110468926	110466926:110469874	AATGTAGGCCAGAGTTGA	ups	bckwd	52	110468873
Otx2	18424	orthodenticle homolog 2	chr14:-:49282547	49280547:49284547	AAAATGGGACATAGTTCA	dns	bckwd	966	49281580
Pcbp3	59093	poly(rC) binding protein 3	chr10:-:76424692	76422692:76426692	GAAGTGGGTTGAGGGTGA	ups	bckwd	1087	76425780
Pcnt	18541	pericentrin (kendrin)	chr10:-:75905657	75903657:75907657	GAAGTGGGTGCAGGGTGA	dns	bckwd	1301	75904355
Pex16	18633	peroxisomal biogenesis facto	chr2:+:92215395	92213395:92217395	GAAATGGGTCAAAGGTCA	dns	bckwd	446	92215842
Pfkm	18642	phosphofructokinase, muscle	chr15:+:97923019	97921019:97925019	AAACTGGGGGAGGGGTGA	dns	bckwd	661	97923681
Pias2	17344	protein inhibitor of activated	chr18:+:77304418	77302418:77306418	AATATAGGTGAAAGTTTA	dns	bckwd	1543	77305962
Piwil4	330890	piwi-like RNA-mediated gene	chr9:-:14545177	14543177:14547177	AATATGGGTGAGGGGTTA	ups	forwd	1926	14547104
Plekha1	101476	pleckstrin homology domain	chr7:+:138009423	138007423:138011423	GATGTGGGGGGTGGGTGA	ups	forwd	1672	138007750
Plekhj1	78670	pleckstrin homology domain	chr10:-:80261371	80259371:80263371	AAACTGGGGCCTGGGTGA	ups	forwd	1258	80262630
Pmm1	29858	phosphomannomutase 1	chr15:-:81791360	81789360:81793360	GATCTAGGGCAAGGTTTA	ups	bckwd	1105	81792466
Pnpla2	66853	patatin-like phospholipase do	chr7:+:148641086	148639086:148643086	GAAGTAGGTCAGAGTTGA	dns	forwd	1733	148642820
Ppapdc1b	71910	phosphatidic acid phosphata	chr8:+:26830519	26828519:26832519	AAACTGGGCTGAGGGTGA	ups	bckwd	287	26830231
Ppef1	237178	protein phosphatase with EF	chrX:-:157157904	157155904:157159904	TATGTAGGCTGAAGTTGA	dns	forwd	282	157157621
Ppp1r3f	54646	protein phosphatase 1, regul	chrX:-:7151407	7149407:7153407	TAAATAGGGCTAAGGTTA	dns	forwd	1324	7150082
Psmd11	69077	proteasome (prosome, macr	chr11:+:80242116	80240116:80244116	AAAATAGGTTAAGGTTCA	dns	bckwd	1753	80243870
Ptger2	19217	prostaglandin E receptor 2 (s	chr14:+:45607785	45605785:45609785	AATATAGGTGAAGGTTGA	ups	bckwd	1840	45605944
Ralgds	19730	ral guanine nucleotide dissoc	chr2:+:28368686	28366686:28370686	GAAATGGGGTCAGGGTCA	dns	forwd	1270	28369957
Rbck1	24105	RanBP-type and C3HC4-type	chr2:-:152158161	152156161:152160161	AATATAGGTAAAGGGTCA	ups	bckwd	960	152159122
Rbck1	24105	RanBP-type and C3HC4-type	chr2:-:152158375	152156375:152160375	AATATAGGTAAAGGGTCA	ups	bckwd	746	152159122
Rpl3l	66211	ribosomal protein L3-like	chr17:+:24864773	24862773:24866773	AATGTGGGTGAGAGGTTA	dns	forwd	1791	24866565
Rpn1	103963	ribophorin I	chr6:+:88034466	88032466:88036466	TAAGTGGGCAAAAGTTTA	ups	bckwd	272	88034193
Rps12	20042	ribosomal protein S12	chr10:-:23507015	23505015:23509015	TAAGTGGGCTAGAGTTGA	dns	bckwd	587	23506427
Rps5	20103	ribosomal protein S5	chr7:+:13507659	13505659:13509659	AATATGGGCCATAGGTTA	ups	bckwd	1598	13506060
Rtn4	68585	reticulon 4	chr11:+:29618562	29616562:29620562	AATGTGGGGGAAAGGTGA	dns	forwd	1244	29619807
Sema3b	20347	sema domain, immunoglobu	chr9:-:107511572	107509572:107513572	GATGTGGGGGAGGGGTTA	ups	forwd	444	107512017
Sema6c	20360	sema domain, transmembrar	chr3:+:94968260	94966260:94970260	AATATGGGTGAGGGGTTA	dns	forwd	1386	94969647
Senp1	223870	SUMO1/sentrin specific pept	chr15:-:97924000	97922000:97926000	AAACTGGGGGAGGGGTGA	dns	forwd	301	97923698
Serinc2	230779	serine incorporator 2	chr4:-:129952830	129950830:129954830	GAAGTGGCCGAGGTTCA	ups	forwd	1625	129954456
Sestd1	228071	SEC14 and spectrin domains	chr2:-:77118649	77116649:77120649	GAAGTGGGAGATGGGTGA	ups	forwd	380	77119030
Sf3a2	20222	splicing factor 3a, subunit 2	chr10:+:80261479	80259479:80263479	AAACTGGGGCCTGGGTGA	ups	bckwd	1150	80262630
Shroom4	208431	shroom family member 4	chrX:+:5977262	5975262:5979262	AAAGTGGGTTAAAGTTCA	dns	bckwd	1048	5978311
Slc41a2	338365	solute carrier family 41, mer	chr10:-:82800562	82798562:82802562	AAACTGGGCCATAGTTCA	ups	forwd	1758	82802321
Slc52a3	69698	solute carrier protein family	chr2:+:151825601	151823601:151827601	GAACTAGGGGAAAGGTCA	dns	bckwd	130	151825732
Smarca1	93761	SWI/SNF related, matrix asso	chrX:-:45245729	45243729:45247729	GAAGTGGGGCAGGGTTGA	ups	forwd	1	45245731
Smpdl3a	57319	sphingomyelin phosphodiest	chr10:+:57514349	57512349:57516349	TAAGTGGGCGTTGGTTGA	dns	bckwd	1882	57516232
Snx27	76742	sorting nexin family member	chr3:-:94386638	94384638:94388638	AAAGTGGGTGGAGGGTTA	dns	bckwd	907	94385730
Sparc	20692	secreted acidic cysteine rich	chr11:-:55233582	55231582:55235582	TATCTGGGGCAGGGGTGA	dns	bckwd	1956	55231625
Spg7	234847	spastic paraplegia 7 homolog	chr8:+:125589407	125587407:125591407	AAACTGGGCTGTGGGTGA	dns	forwd	582	125589990
Sphkap	77629	SPHK1 interactor, AKAP dom	chr1:-:83404775	83402775:83406775	GATGTAGGTAAAAGTTCA	dns	forwd	1735	83403039
Spink13	1E+08	serine peptidase inhibitor, Ka	chr18:-:62901041	62899041:62903041	TAAGTAGGCAAAAGGTGA	dns	bckwd	398	62900642
Sptbn1	20742	spectrin beta, non-erythrocy	chr11:-:30098257	30096257:30100257	GAAATAGGTGCAGGGTGA	dns	bckwd	255	30098001
Srsf4	57317	serine/arginine-rich splicing	chr4:+:131429553	131427553:131431553	AAAATGGCCCAAGGTCA	ups	forwd	325	131429227

Ssr2	66256	signal sequence receptor, be	chr3:+:88383592	88381592:88385592	AAAATGGGGGGTAGGTCA	ups	forwd	1273	88382318
Ssu2	243612	ssu-2 homolog (C. elegans)	chr6:-:112338017	112336017:112340017	AATCTGGGGCTTAGTTCA	ups	forwd	638	112338656
Stpg1	78806	sperm tail PG rich repeat cor	chr4:+:135051901	135049901:135053901	GAACTGGGCGGTGGGTGA	dns	forwd	274	135052176
Sult2a3	629203	sulfotransferase family 2A, d	chr7:-:14708342	14706342:14710342	TATATAGGCCAGAGTTCA	dns	forwd	1647	14706694
Suv39h1	20937	suppressor of variegation 3-9	chrX:-:7651439	7649439:7653439	TATGTAGGTCAAAGGTCA	ups	forwd	1144	7652584
Suv39h1	20937	suppressor of variegation 3-9	chrX:-:7651886	7649886:7653886	TATGTAGGTCAAAGGTCA	ups	forwd	697	7652584
Tbc1d8	54610	TBC1 domain family, membe	chr1:-:39535592	39533592:39537592	AATATGGGTGAGAGGTGA	ups	bckwd	1861	39537454
Tbx22	245572	T-box 22	chrX:+:104874353	104872353:104876353	AATCTGGGTTCAAGGTGA	ups	forwd	169	104874183
Tceal1	237052	transcription elongation fact	chrX:+:133242603	133240603:133244405	TAAGTAGGCTAAAGGTTA	ups	forwd	1483	133241119
Tfb1m	224481	transcription factor B1, mito	chr17:-:3557713	3555713:3559713	TATATAGGGTGAAGGTCA	dns	forwd	634	3557078
Tmc2	192140	transmembrane channel-like	chr2:+:130020929	130018929:130022929	TATCTGGGCTTTGGTTTA	dns	bckwd	918	130021848
Tmco2	69469	transmembrane and coiled-c	chr4:-:120781831	120779831:120783831	AAACTGGGGTAGGGGTTA	dns	bckwd	1606	120780224
Tmed9	67511	transmembrane emp24 prot	chr13:+:55694495	55692495:55696495	AATCTGGGGCAGGGTTGA	dns	bckwd	1387	55695883
Tmem135	72759	transmembrane protein 135	chr7:-:96487297	96485297:96489297	AATCTGGGGCAGAGTTCA	dns	bckwd	1648	96485648
Tmem140	68487	transmembrane protein 140	chr6:+:34813145	34811145:34815145	TAACTGGGTCTTGGTTCA	ups	bckwd	661	34812483
Trhr	22045	thyrotropin releasing hormo	chr15:+:44027680	44025680:44029680	TAAGTAGGGCAAAGTTGA	dns	bckwd	1804	44029485
Txndc11	106200	thioredoxin domain containi	chr16:-:11088023	11086023:11090023	TATATGGGTCAGGGGTCA	ups	forwd	1674	11089698
V1ra8	113850	vomeronasal 1 receptor, A8	chr6:+:90152810	90150810:90153650	AAAATGGGACAAGGTTCA	ups	bckwd	1625	90151184
Wdr62	233064	WD repeat domain 62	chr7:-:31065440	31063440:31067440	GATATAGGGGTAGGGTGA	ups	bckwd	269	31065710
Zbtb40	230848	zinc finger and BTB domain c	chr4:-:136604610	136602610:136606610	AATGTAGGGTTAGGGTTA	dns	forwd	987	136603622
Zfp444	72667	zinc finger protein 444	chr7:+:6124114	6122114:6126114	GAACTGGGTAAAGGTTGA	dns	forwd	1822	6125937
Zfp667	384763	zinc finger protein 667	chr7:+:6238181	6236181:6240181	TAACTGGGGGAAAGGTGA	dns	bckwd	1792	6239974
Zfp804a	241514	zinc finger protein 804A	chr2:+:81893814	81891814:81895814	GATATGGGTGGAAGGTGA	dns	forwd	868	81894683
